# Imported Schistosomiasis in Southwestern Europe: Wide Variation of Pure and Hybrid Genotypes Infecting Sub-Saharan Migrants

**DOI:** 10.1155/tbed/6614509

**Published:** 2025-04-18

**Authors:** Alejandra De Elias-Escribano, Patricio Artigas, Joaquin Salas-Coronas, María Pilar Luzon-Garcia, Marta Reguera-Gomez, Raquel Sanchez-Marques, Fernando Salvador, Jerôme Boissier, Santiago Mas-Coma, Maria Dolores Bargues

**Affiliations:** ^1^Department of Parasitology, Faculty of Pharmacy, University of Valencia, Burjassot, Valencia, Spain; ^2^Networked Biomedical Research Center for Infectious Diseases (CIBERINFEC), Carlos III Health Institute, Madrid, Spain; ^3^International Health Research Group of Almería (GISIA), Faculty of Health Sciences, University of Almería, Almería, Spain; ^4^Tropical Medicine Unit, Poniente University Hospital, El Ejido, Almería, Spain; ^5^Tropical Medicine Unit Vall d'Hebron-Drassanes, Infectious Diseases Department, Vall d'Hebron University Hospital, PROSICS Barcelona, Barcelona, Spain; ^6^Hosts Pathogens Environment Interactions, UMR 5244, CNRS, IFREMER, Perpignan University, Via Domitia, Perpignan, France

**Keywords:** imported schistosomiasis, mitochondrial (*cox*1) and nuclear (ITSs and 18S) markers, pure and hybrid infections (haplotype complexity), RD-PCR and sequencing, Spain

## Abstract

Mitochondrial *cox*1 and nuclear ribosomal ITSs are commonly combined to distinguish *Schistosoma* species and identify hybrids in endemic countries but very rarely applied to patients diagnosed in Europe despite the increasing arrival of migrants in southwestern Europe. To assess whether those migrants are carriers of pure or hybrid schistosomes, a complete genetic characterization of *Schistosoma* entering Spain is performed. A total of 759 eggs (from urine + stools) from 58 patients from 8 African countries were individually processed to describe their mito-nuclear signature by *cox*1 rapid diagnostic multiplex one-step polymerase chain reaction (RD-PCR) and ITS-2/18S sequencing and haplotype identification by means of the complete ITS1-5.8S-ITS2 rDNA and *cox*1 sequencing. Combined nuclear and mitochondrial DNA markers in sub-Saharan migrants residing in Spain are described for the first time. Twenty-two (40.74%) patients were simultaneously carrying pure and hybrid eggs in their urine. S*chistosoma haematobium* × *S. bovis* (68.18%) and *S. haematobium* × *S. curassoni* (31.82%) hybrid combinations were the most frequent. Six (one pure and five hybrid) and two (pure) mito-nuclear signatures, in urine and stools, respectively, and 12 nuclear and 61 mitochondrial imported haplotypes were found. This study highlights the genetic complexity of pure and hybrid schistosomes that enter Spain, and consequently Europe, and contributes to the following: correlate the geographical origin of patients with pure and/or hybrid genetic types; detect the presence of hybrids “at distance” (hybrids in migrants from Guinea-Bissau and Mauritania are first time detected); correlate molecular haplotypes with pathologies, clinical pictures, and treatment responses; and, importantly, warn about possible sources of autochthonous transmission.

## 1. Introduction

Schistosomiasis is one of the most important neglected tropical diseases due to its great socioeconomic public health impact. This chronic and debilitating disease primarily affects impoverished communities lacking access to safe drinking water and adequate sanitation in tropical and subtropical regions, with the highest burden on the African continent [1].

It is caused by a dioecious trematode of the genus *Schistosoma* with a two-host life cycle, combining asexual multiplication larval processes in the intermediate freshwater snails and sexual reproduction in the definitive mammalian host [2, 3]. Each schistosome species shows specificity for both its snail and mammalian hosts, which heads its distribution [4]. In the sub-Saharan Africa, four out of the six species that infect humans have been reported, *Schistosoma mansoni*, *S. intercalatum*, and *S. guineensis*, which are responsible for intestinal schistosomiasis, and *S. haematobium*, the only species causing urogenital schistosomiasis. In addition to its medical importance, schistosomiasis also has a major impact on livestock morbidity and mortality. Species in Africa that have received particular attention because of their veterinary impact in ruminants are *S. bovis*, *S. curassoni* and/or *S. mattheei* [5, 6].

Natural hybridization between closely related species is increasingly reported in schistosomes. Hybrids of *S. haematobium* with livestock-infecting species are of particular interest, as the causative agent of urogenital schistosomiasis has always been considered an exclusively human-specific parasite [2, 7, 8]. Previously geographically separated species may now co-occur in the same area due to the constant movement of humans, livestock, and wildlife, as well as environmental changes. Possibilities of hybridization are strongly increased in schistosomes because they have separate sexes and need to mate to reproduce sexually in the definitive host [6, 9].

In nonendemic countries, the disease is expected to be rarely diagnosed and only associated with international travel and migratory movements. This has been the case in Europe since the disease was eradicated in Portugal and Greece in the last century [3]. Nevertheless, in 2014, urogenital schistosomiasis cases were detected in European patients who had never traveled to an endemic area. The infection, which reached up to 106 confirmed cases, had been acquired in the Cavu River, a popular tourist bathing area in southern Corsica (France) [10, 11], and was caused by a *S. haematobium*–*S. bovis* hybrid strain imported from Senegal [12]. The disease has become established on this Mediterranean island, as subsequent cases have been recently detected in the Solenzara River in the southeastern part of the island [13, 14]. The persistence of this infection appears related to the ability of *Bulinus truncatus* intermediate snails to overwinter with the infection and produce viable cercariae once optimal temperatures are achieved [15]. One of the *B. truncatus* strains used in the aforementioned study originated from a locality of the Poniente area in Spain, where autochthonous human urogenital schistosomiasis cases have recently been reported [16].

Spain, like many Mediterranean regions, is a susceptible country where there is a risky coexistence of a competent intermediate host [17–19] and numerous potential schistosome-infected migrants and travelers returning from sub-Saharan Africa. This mainly concerns the country's southern region, where schistosomiasis is one of the most prevalent diseases diagnosed in sub-Saharan migrant populations [20–23].

The most commonly reported schistosome infections in Spain are caused by *S. mansoni* and *S. haematobium* [22, 24–26], the two species that represent the highest burden of infection in sub-Saharan Africa, with some sporadic reports of *S. intercalatum* [21, 27], also present on the African continent. Species classification in Spanish patients, as in most reports from other European countries, is based only on the lateral or terminal position of the egg spine and the egg excretion way by urine or feces. These two characteristics do not allow to detect infections produced by hybrids [28], which are becoming increasingly common in Africa, among which *S. haematobium* hybrids with livestock schistosomes of high veterinary importance stand out due to their potential zoonotic transmission [5, 6].

Increased people movements, including migration, along with the widespread *S. haematobium* susceptible planorbid species in Mediterranean countries, raise the likelihood of urogenital schistosomiasis spreading further in Europe. Indeed, the presence of *Bulinus* spp. has been reported in Cyprus, France, Greece, Italy, Spain, and Portugal [10, 18, 29–31]. In Spain and Portugal, there is also the *S. bovis* transmitter snail *Planorbarius metidjensis*, whose local wild populations were found to be naturally infected wit*h S. haematobium* at the time of the old schistosomiasis outbreak in the 1920s [32–35]. All these countries suffer intense migratory flows from African countries. Moreover, climate change could contribute to the expansion of snail distribution areas, facilitating their adaptation to new environmental conditions. Additionally, snails could spread through the trade of aquatic plants or bird migrations [19, 36].

Here, we aim to describe the genetic profile of imported schistosomes arriving to Spain with an increasing migrant population coming from highly endemic countries. For this genetic characterization, the sequences of the molecular markers of the nuclear ribosomal DNA (ITSs and 18S) and the mitochondrial DNA (*cox*1) are used. The genetic strains of *Schistosoma* that enter Spain, and consequently Europe, provide a baseline for the clinical monitoring of pure or hybrid infections, along with the warn of the potential risk of schistosomiasis introduction into European nonendemic areas.

## 2. Materials and Methods

### 2.1. Filtration of Urine and Stool Samples

**Figure 1:**
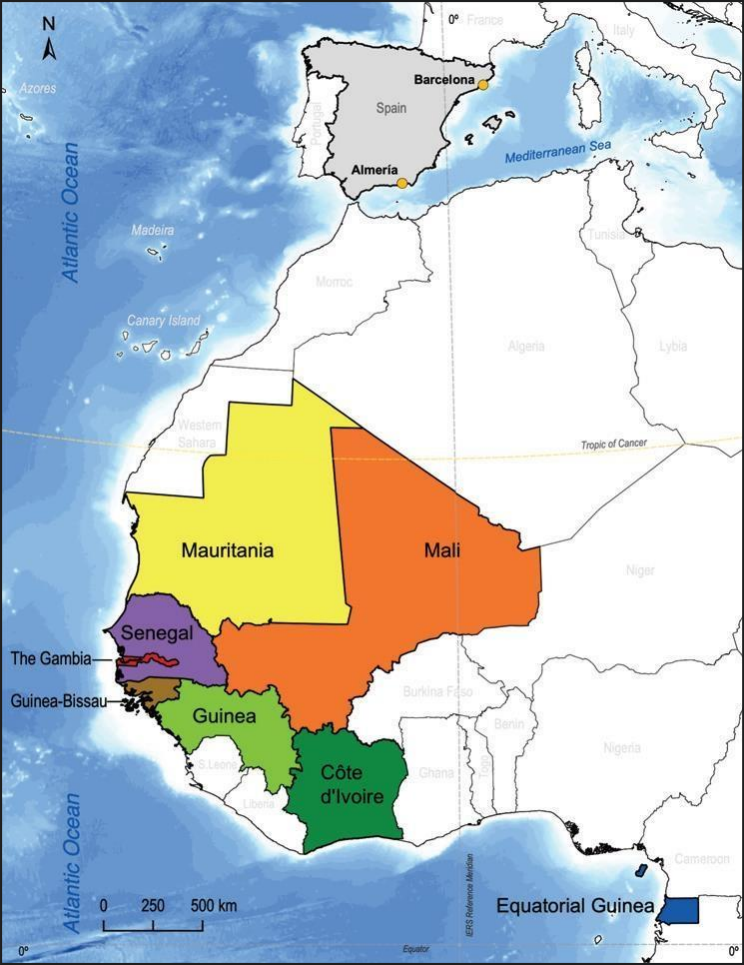
Map of Africa with the countries of origin (in color) of migrants diagnosed in Spain (Almería and Barcelona) with schistosomiasis and included in this study.

A total of 759 schistosome eggs collected from 58 migrants, 57 males and one female (aged 17–52 years, average 26.07), with a mean residence in Spain of 20.89 (1–192) months, were analyzed (Table 1). The migrants came from eight different sub-Saharan African countries (Figure 1), with Mali (*n* = 24), Senegal (*n* = 18), and Mauritania (*n* = 8) as the most frequent countries of origin. All patients were diagnosed and treated at the Tropical Medicine Unit (TMU) of Hospital Universitario de Poniente (El Ejido, Almería, Spain) and at the TMU of Vall d'Hebron University Hospital, Barcelona (Spain), between October 2016 and June 2023.

Parasite eggs from each urine sample were obtained by using a sterilized nylon cell filter with a pore size of 40 µm (Falcon, Durham, NC, USA) and 0.9% NaCl saline solution to wash the sample through the filter. All eggs trapped on the strainer were collected in a Petri dish containing 20 mL of 0.9% saline solution. Stool samples were individually filtered, using the same saline solution, through a column of metal sieves, arranged in decreasing order of pore size (0.5, 0.25, 0.125, 0.1, and 0.04 mm) (FILTRA, Barcelona, Spain) to isolate parasite eggs from the debris. All eggs trapped on the last sieve were recovered in a Petri dish.

After filtering the urine and stool samples, the eggs were individually picked up with a 10 μL pipette from the Petri dishes under a stereomicroscope. Each egg was then transferred to an Eppendorf vial containing 50 µL of 70% ethanol for subsequent individual molecular characterization.

Only urine or fecal samples from patients with confirmed schistosomiasis, in which the presence of *Schistosoma* eggs in urine or feces was demonstrated, were included in this study.

### 2.2. Molecular Methods

#### 2.2.1. DNA Extraction

Genomic DNA was extracted individually from each of the 759 eggs (708 terminal-spine eggs and 51 lateral-spine eggs) recovered from 54 urine and 5 stool samples (Table 1) using the InstaGene Matrix kit (Bio-Rad Laboratories CA, USA) according to the manufacturer's instructions. The resulting supernatant (90–95 μL), containing the extracted DNA, was subsequently stored at −20°C for further analysis.

#### 2.2.2. Rapid Diagnostic Multiplex One-Step Polymerase Chain Reaction (RD-PCR) of *cox*1

To identify the species-specific mitotype of each sample, a RD-PCR of the cytochrome c oxidase subunit I (c*ox*1) of the mitochondrial DNA (mtDNA) was performed from each of the 759 eggs, with either terminal-spined or lateral-spined eggs according to their morphology (Table 2). For this purpose, a single universal forward primer and three species-specific reverse primers amplifying different length for *S. bovis* (306 bp), *S. mansoni* (375 bp), and *S. haematobium* (543 bp) were used. Primers and PCR conditions were as previously described [37–40]. The *cox*1 RD-PCR products were visualized by electrophoresis on a 2.5% agarose gel stained with GelRed (Biotium) and photographed using the UVP gel documentation system. All eggs that presented a *S. bovis* or *S. curassoni* band were subsequently identified by *cox*1 sequencing, as RD-PCR cannot differentiate between *S. bovis* and *S. curassoni* [2, 41].

#### 2.2.3. rDNA and mtDNA PCR Amplification and Sequencing

The information provided by the maternally inherited mtDNA profiling by RD-PCR was complemented with the partial sequence of the 5.8S rRNA gene and the complete internal transcribed spacer 2 (ITS-2) of the rDNA, to create a mito-nuclear signature, similar to what has been carried out in previous research [7, 42]. PCR amplification was performed independently on each of the 759 eggs using primers 3S and A28S [39, 40, 43–45], which partially amplified the 5.8S gene (139 bp) and the entire ITS-2 (313 bp). The PCR was programmed for an activation step of 4 min at 94°C, continued by 32 cycles of 55 s at 94°C, 1 min at 55–62°C and 1.30 min at 72°C each and a final extension of 5 min at 72°C followed by a final cooling at 4°C.

To resolve ambiguities caused by double peaks at four polymorphic positions in the ITS-2 sequence, which do not differentiate between *S. bovis* (*Sb*) and *S. curassoni* (*Sc*), additional amplification and sequencing were conducted for the 5′ end of the nuclear ribosomal 18S DNA gene (1369 bp). The primers and PCR conditions were carried out as previously described [46], with an initial step of 3 min at 94°C, followed by 35 cycles of 30 s at 94°C, 40 s at 50°C, and 1.50 min at 72°C each, ending with a 7-min cycle at 72°C.

A molecular haplotype identification process was conducted on a randomly selected subset of 285 eggs (4–6 eggs/patient), comprising 251 from urine and 34 from stool samples (Table 2). These samples represented migrants from eight countries diagnosed with urinary or intestinal schistosomiasis. The analysis involved amplification and sequencing of the complete ITS1-5.8S-ITS2 (ITS) region (927 bp) and partial *cox1* mitochondrial DNA (1024–1052 bp). PCR amplifications were carried out with primers BD1 and BD2 [47], for the ITS region, following PCR conditions previously described [39, 43, 45], and primers Cox1_schist F and Cox1_schist R [48], for *cox*1, using PCR conditions as previously described [3].

PCR amplifications of ITS-2, 18S, ITS1-5.8S-ITS2, and *cox*1 (long fragment) were conducted using a Veriti 96-well thermal cycler (Applied Biosystems, Thermo Fisher Scientific, Waltham, MA, USA). The resulting PCR products were then purified with the Ultra Clean PCR Clean-up DNA Purification System (MoBio, Solana Beach, CA, USA) following the manufacturer's instructions and resuspended in 50 μL of 10 mM TE buffer (pH 7.6). The final DNA concentration (in µg/mL) and the 260/280 nm absorbance ratio were measured using an Eppendorf BioPhotometer (Eppendorf, Hamburg, Germany).

Each of the DNA markers was independently amplified by PCR for each egg, and the resulting PCR products were sequenced on both strands by the dideoxy chain termination method. This was carried out using the Taq dye-terminator chemistry kit on an Applied Biosystems 3730xl DNA Analyzer (Applied Biosystems, Foster City, CA, USA) using the same amplification PCR primers.

#### 2.2.4. Sequence Analyses

Forward and reverse sequences of the ITS-2, 18S, ITS1-5.8S-ITS2, and *cox*1 (long fragment) markers were edited and assembled for each individual DNA marker using Sequencher v. 5.4.6 (Gene Codes Co., MI, USA). The alignment of these sequences was performed with ClustalW using default parameters in the MEGA X software [49]. Penalties for gaps were applied in both pairwise and multiple sequence alignments. The divergence of sequences within and among each marker was measured by the total character differences. All changes, including transitions (ts), transversions (tv), and insertions/deletions (indels), were treated as character states in MEGA X.

For the ITS rDNA sequences, a thorough examination of all nucleotide positions in the raw sequence chromatograms was performed to detect sequence polymorphisms between *S. haematobium* and *S. bovis* or *S. curassoni*. This process was used to identify any possible heterozygosity, especially at the polymorphic positions that differentiate these species, as previously documented [2, 42, 50]. In the case of the 18S rDNA sequence, five single-nucleotide polymorphisms (SNPs) allowing the distinction between *S. haematobium*, *S. bovis* and *S. curassoni* [51] were carefully checked for final species adscription (Table S1). The reference sequences used from GenBank were *Sh* from Mali and Tanzania (Z11976 and OX103963); *Sb* from Kenia (OX104095); and *Sc* from Senegal (AY157236), for the 18S gene, and *Sh* from Tanzania (OX103963), *Sb* from Kenia (OX104095), and *Sc* from Senegal (MT580946), for the ITSs.

The aligned sequences were collapsed into haplotypes using the ALTER web server [52], with gaps counted as differences. Homologies were determined using the BLAST program from the National Center for Biotechnology Information website (http://www.ncbi.nlm.nih.gov/BLAST). Complete or almost complete sequences (with similar length in bp of the same molecular markers used in this study, 100% coverage and greater than 99% similarity in BLAST with our sequences) were retrieved from GenBank for sequence analysis comparisons and haplotype identification (Tables S2 and S3).

#### 2.2.5. Mito-Nuclear Code Nomenclature for Species and Hybrid Identification

For mito-nuclear signature, we used RD-PCR of *cox*1 and ITS-2 sequencing analysis to determine species and identify hybrids. The nomenclature utilized here is the same as that used in previous studies [7, 42], assuming that the *cox*1 gene is a mitochondrial marker of haploid inheritance, so only one allele is indicated by the abbreviation of the species involved. Whereas ITS-2 is a marker of biparental inheritance, each allele is indicated by the two-letter abbreviation of the identified species. For instance, the mito-nuclear signature for defining the genetic profile of “pure” *S. haematobium* will be *S. haematobium cox*1 × *S. haematobium* ITS-2 = *ShxShSh*, in which species identification is indicated by two-letter abbreviation (e.g., *S. haematobium* = *Sh*).

Using this mito-nuclear code, we defined hybrid eggs when the *cox*1 and ITS-2 markers are read together, and the results are discordant in species assignment (e.g., *S. bovis cox*1 × *S. haematobium* ITS-2 = *SbxShSh*) and/or when heterozygous positions are observed in the ITS-2 sequences at points that discriminate between species (e.g., *S. haematobium cox*1 × *S. haematobium* × *S. bovis* ITS-2 = *ShxShSb*).

In the specific case of *S. haematobium*, *S. bovis*, and *S. curassoni*, the ITS-2 allows differentiation between *S. haematobium* and *S. bovis* or *S. curassoni* but not between *S. bovis* and *S. curassoni*. Therefore, when obtaining *S. haematobium cox*1 × *S. haematobium* × *S. bovis/curassoni* ITS-2, the 18S marker was used for species confirmation. The code *ShxShSb/Sc* was used only in cases where there was insufficient DNA for 18S sequencing.

If no discordance is observed in mito-nuclear signature, eggs are considered pure.

#### 2.2.6. Haplotype Code Nomenclature

The haplotype nomenclature used is organized by first identifying the species with a two-letter abbreviation or, in the case of hybrids, the species involved (as in the case of the mito-nuclear signature), second the genetic marker used (ITSs or *cox*1), and third the haplotype (H) number (1, 2, 3, etc.). In the case of ribosomal hybrid haplotypes, these are indicated by Htz. According to that, a pure *S. haematobium* haplotype from the complete intergenic region sequencing would be Sh-ITSs-H1, and a hybrid *S. haematobium* × *S. curassoni* haplotype would be ShxSc-ITSs-Htz1, and an example of a pure *S. haematobium* haplotype from partial sequencing of *cox*1 would be Sh-*cox*1-H1.

### 2.3. Phylogenetic Analyses

Molecular phylogenetic analyses were carried out by means of haplotype networks and phylogenetic trees.

Haplotype networks were generated to depict relationship among *S. haematobium*, *S. bovis*, and *S. mansoni*, detected in migrant people in Spain and other southwestern Europe and African and American samples with Network 10.2.0.0. (http://www.fluxus-engineering.com/) software. The three independent networks (one for each *Schistosoma* species) were constructed with the sequences obtained of *cox*1 mtDNA and other sequences retrieved from GenBank with a length similar to those obtained in this study (>1000 bp) (Table S4), using median-joining (MJ) network algorithm, with default parameters (equal character weight = 10, transitions/transversions weight = 1:1, epsilon = 0, frequency >1, and connection cost as a criterion), in Network. Hypothetical median vectors (a hypothesized sequence that is required to connect existing sequences within the network with maximum parsimony) were added to the network for shortest connection between the data set.

Phylogenetic tree was carried out including all the *S. haematobium*, *S. bovis*, *S. guineensis*, and *S. mansoni cox*1 haplotypes identified in this study (61) plus 22 reference haplotypes/isolates obtained from GenBank Nucleotide Database (accession numbers are shown in the tree, results Section 3.5) and *S. japonicum* (KU196417) as outgroup. The initial data matrix contains 84 sequences that were collapsed into 76 haplotypes and 1024 positions in the final data set. The best substitution model selection analysis was run in MEGA X, considering the Bayesian information criterion (BIC) scores; the Akaike information criterion, corrected (AICc) value; the maximum likelihood (ML [lnL]) value; and the number of parameters (including branch lengths) for each model. The evolutionary history was inferred by using the ML method. The initial tree(s) for the heuristic search were automatically obtained using the nearest-neighbor interchange (NNI) method by applying the neighbor-joining and BioNJ algorithms to a matrix of pairwise distances estimated using the maximun composite likelihood (MCL) approach and then selecting the topology with the superior log likelihood value. To assess the reliability of the nodes in the trees, a bootstrap analysis using 1000 replicates was made using Bootstrap method in MEGA X.

## 3. Results

### 3.1. Genetic Signature

Out of the 759 eggs examined, the complete mito-nuclear signature was obtained for 702 of them, of which 642 from urine and 60 from feces (Table 3).

Of the 642 terminal-spined eggs from urine, 558 (86.92%) exhibited by RD-PCR an *S. haematobium cox*1 profile, while 84 (13.08%) showed an *S. bovis* (or *S. curassoni*) profile. Regarding ITS-2 sequences, 612 (95.33%) yielded an ITS-2 corresponding to *S. haematobium*, while 30 (4.67%) showed alleles of *S. haematobium* and *S. bovis* or *S. haematobium* and *S. curassoni* in their resulting ITS-2 and 18S sequences (Table 3).

When the *cox*1 and ITS-2/18S markers were considered together, six mito-nuclear signatures were obtained: (i) *ShxShSh* (83.33%), corresponding to pure *S. haematobium*; (ii) *ShxShSc* (2.80%), resulting from a hybrid between *S. haematobium* and *S. curassoni*; (iii) *SbxShSh* (11.99%), *SbxShSb* (0.32%), and *ShxShSb* (0.47%), belonging to *S. haematobium* × *S. bovis* hybrid combinations; and (iv) *SbxShSc* (0.78%) resulting from the interaction of the three species, *S. haematobium* × *S. bovis* × *S. curassoni* (Table 3). Unfortunately, only two hybrid eggs remained as *ShxShSb/Sc*, as there was no more DNA available for 18S rRNA sequencing.

Out of the 60 eggs from stools, RD-PCR successfully identified the *S. mansoni cox*1 profile in 51 lateral-spined eggs. No *S. haematobium* or *S. bovis cox*1 profiles were detected. Furthermore, the RD-PCR was nonspecific with primers used (*S. haematobium*, *S*. *bovis, and S. mansoni*) for the nine terminal-spined eggs, which were later identified as *S. guineensis* by *cox*1 and ITS-2 sequencing. As a result, their signature was determined to be *SgxSgSg* (Table 3). Genetic profiles based on their mito-nuclear signature were coincident with the morphology of the eggs. While the 51 lateral-spined eggs provided a profile of *S. mansoni* in both *cox*1 and ITS-2 markers, giving a *SmxSmSm* signature, the nine terminal-spined eggs presented a *S. guineensis* profile with an *SgxSgSg* signature. No hybrid mito-nuclear signature was detected in stool samples.

A summary of genetic profiles of both urine and stool genotyped eggs is listed in Table 3.

### 3.2. Genetic Identification of Pure/Hybrid Infections According to Patients and Country of Origin

The analysis of 58 patients allowed us to identify 36 (62.07%) carriers of “pure” eggs and 22 (37.93%) carriers of “pure” and hybrid eggs.

Among the 54 patients with terminal-spined eggs in their urine, 32 (59.26%) were clasified as carriers of “pure” *S. haematobium* and corresponding to the mito-nuclear signature *ShxShSh*. These patients came from Gambia, Guinea-Bissau, Côte d'Ivoire, Mali, Mauritania, and Senegal. The remaining 22 patients (40.74%) were identified as simultaneous carriers of pure *S. haematobium* (*ShxShSh*) and hybrid eggs in their urine. The different hybrid mito-nuclear signatures obtained by patient are detailed in Table 4.

Among the five patients with lateral-spined or terminal-spined eggs in their stool samples, four of them, Côte d'Ivore (one), Guinea (one), and Senegal (two), were carriers of pure *S. mansoni* (*SmxSmSm*) and the fifth, from Equatorial Guinea, was infected with *S. guineensis* (*SgxSgSg*). It is noteworthy that one of these patients from Senegal did also present terminal-spined eggs in their urine and therefore showed a *ShxShSh* mito-nuclear signature, in which no ectopic or hybrid eggs were detected, thus representing a *Sh* + *Sm* coinfection case.

In a single patient from Senegal, two hybrid genetic profiles were detected: *S. haematobium* × *S. bovis* and *S. haematobium* × *S. curassoni* corresponding to the mito-nuclear signatures *SbxShSh* and *ShxShSc*, respectively (Table 4). The remaining patients were carriers of only one type of hybrid genetic profile/patient.

### 3.3. Sequence Analyses for Haplotype Identification

#### 3.3.1. ITS1-5.8S-ITS2 rDNA

**Figure 2:**
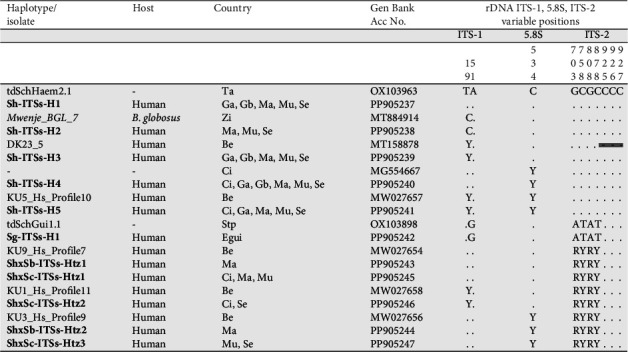
Polymorphic sites in the sequence comparison of the complete transcribed spacer region of the nuclear rDNA between the schistosome haplotypes of terminal-spined eggs from migrants in Spain (in bold) and other haplotypes or isolates of the same, or proximal, species from GenBank. Numbers (to be read vertically) refer to variable positions obtained in the alignment made with MEGA X. . = identical; − = indel; 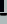 = not sequenced. Heterozygotic positions are represented by the corresponding symbol of IUPAC code for incomplete nucleic acid specification. In the case of identical sequences, only one GenBank accession number has been selected as representative (Table S2 for identities). Be, Benin; Ci, Côte d'Ivoire; Egui, Equatorial Guinea; Ga, The Gambia; Gb, Guinea-Bissau; Ma, Mali; Mu, Mauritania; Se, Senegal; Stp, São Tomé and Principe; Ta, Tanzania; Zi, Zimbabwe.

In the terminal-spined eggs from urine, a total of five haplotypes corresponding to pure *S. haematobium* (Sh-ITSs-H1, Sh-ITSs-H2, Sh-ITSs-H3, Sh-ITSs-H4, and Sh-ITSs-H5) and five hybrid haplotypes were found. The five *S. haematobium* haplotypes showed a 927-bp-long sequence with an average GC content of 49.75%, differing only in two polymorphic sites in positions 19 (T, C or C/T) and 534 (C or C/T) of their alignment (Figure 2). These five haplotypes showed identities with previously described *S. haematobium* isolates or haplotypes available in GenBank database (Figure 2 and Table S2). The five hybrid haplotypes (927-bp-long and 49.5% GC mean content) showed four heterozygous signals at positions 703 (G/A), 758 (C/T), 808 (G/A), and 878 (C/T) in their alignment. After 18S sequencing, three hybrid haplotypes were confirmed as *S. haematobium* × *S. curassoni* (ShxSc-ITSs-Htz1, ShxSc-ITSs-Htz2, and ShxSc-ITSs-Htz3) and two as *S. haematobium* × *S. bovis* (ShxSb-ITSs-Htz1 and ShxSb-ITSs-Htz2). These five hybrid haplotypes also showed identities with previously described haplotypes in human samples from Benin (Figure 2).

In the 10 terminal-spined eggs from stools from the only one patient from Equatorial Guinea, the ITS-1, 5.8S, and ITS-2 region yielded a unique haplotype, identified as Sg-ITSs-H1 (927-bp-long, 49.41% GC), which showed 100% identity with *S. guineensis* (OX103898) reported from São Tomé and Principe.

In the 24 lateral-spined eggs from stools, the ITS-1, 5.8S, and ITS-2 region yielded a unique haplotype shared with patients from Guinea, Senegal, and Côte d'Ivoire. This haplotype Sm-ITSs-H1 (927-bp-long, 48.4% GC) proved to be identical to *S. mansoni* haplotypes/isolates from GenBank and reported from various African countries, plus Brazil, Saudi Arabia, and Oman (Table S2).

#### 3.3.2. *cox*1 mtDNA

The mitochondrial *cox*1 gene sequences from the 251 terminal-spined eggs from urine yielded 39 distinct 1024-bp-long haplotypes, of which 34 displayed a *S. haematobium* profile, while five had an *S. bovis* profile.

**Figure 3:**
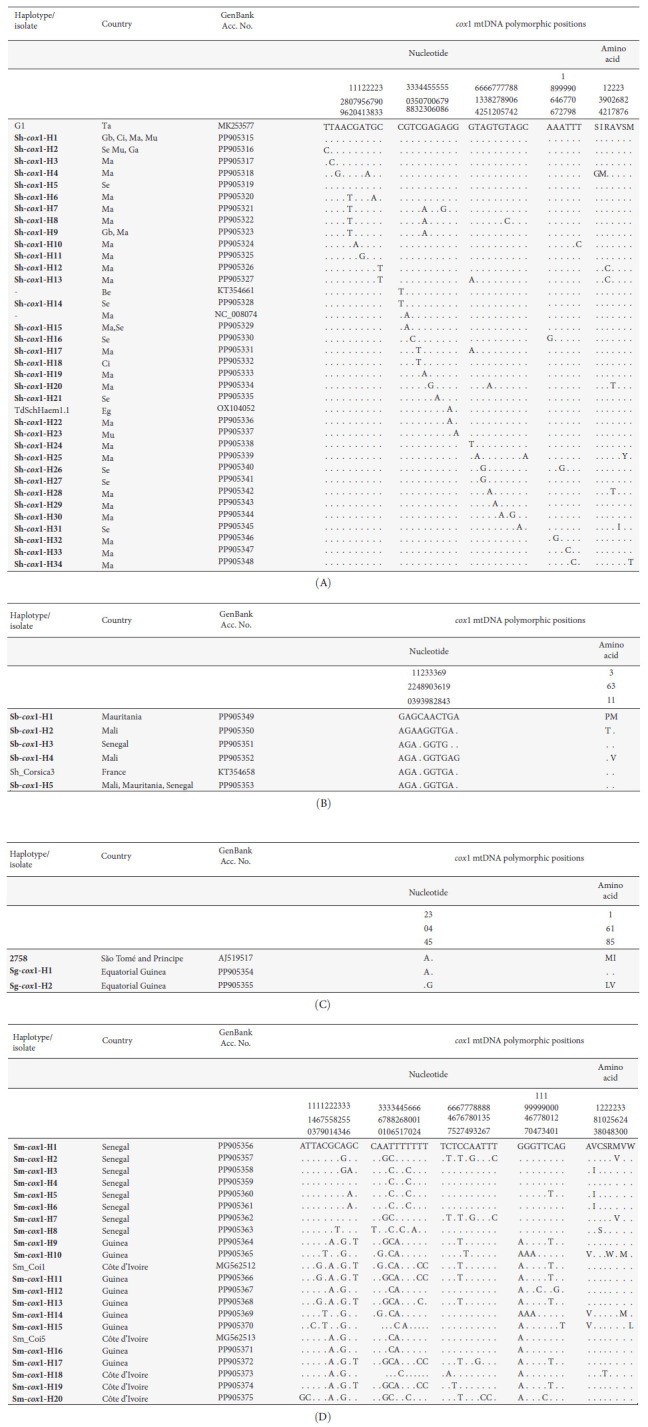
Nucleotide and amino acid polymorphic sites found in the mtDNA *cox*1 sequence of (A) *S. haematobium* (*Sh*), (B) *S. bovis* (*Sb*), (C) *S. guineensis* (*Sg*), and (D) *S. mansoni* (*Sm*) haplotypes (H) (in bold) found in urine and stools of migrant patients. Numbers (to be read vertically) refer to variable positions obtained in the alignment made with MEGA X. . = identical. Be, Benin; Ci, Côte d'Ivoire; Eg, Egypt; Ga, The Gambia; Gb, Guinea-Bissau; Ma, Mali; Mu, Mauritania; Se, Senegal; Ta, Tanzania. In the case of identical sequences (Table S3), only one GenBank accession number has been selected as representative.

The 34 *S. haematobium* haplotypes showed 36 polymorphic sites in their alignment, including 10 parsimony-informative (p-info) and 26 singleton sites, and a mean AT content of 70.24%. These haplotypes were designated as Sh-*cox*1-H1 to Sh-*cox*1-H34. Analysis of the 340-amino-acid-long COX1 protein alignment identified seven variable amino acid positions (Figure 3). Among the 34 newly described haplotypes, only four (Sh-cox1-H1, H14, H15, and H22) exhibited 100% sequence identity with published *S. haematobium* nucleotide sequences available in GenBank (BLAST, 100% coverage; >99% similarity) (Figure 3 and Table S3). The Sh-*cox*1-H1 haplotype proved to be the most common (57.5%) and was detected in patients from Guinea Bissau, Côte d'Ivoire, Mali, Mauritania, and Senegal.

The five *S. bovis* haplotypes showed 10 polymorphic sites in their alignment, including one p-info and nine singleton sites, and a mean AT content of 67.56%. Haplotypes were designated as Sb-*cox*1-H1 to Sb-*cox*1-H5. Analysis of the 340-amino-acid COX1 protein alignment identified two variable amino acid positions (Figure 3). Among the haplotypes, only Sb-*cox*1-H5 showed 100% sequence identity to a previously reported *S. bovis cox*1 haplotype in humans from Corsica (France). This Sb-*cox*1-H5 haplotype was the most common (75.0%) and was detected in patients from Mali, Mauritania, and Senegal (Figure 3).

In the terminal-spined eggs from the stool sample of the Equatorial Guinean patient, two different 1052-bp-long haplotypes were found, Sg-*cox*1-H1 and Sg-*cox*1-H2 (AT content average of 66.44%), differing by only two singleton sites in their alignment. The 350-aa-long COXI protein provided two sequences due to two amino acid changes. The Sg-*cox*1-H1 haplotype was found to be identical to two sequences of *S. guineensis* reported in São Tomé and Principe (Figure 3).

In lateral-spined eggs from the stool samples of patients from Guinea, Senegal, and Côte d'Ivoire, *cox*1 sequencing identified 20 distinct 1024-bp-long haplotypes (AT content average of 66.61%). The alignment of these 20 haplotypes showed 38 variable positions, of which 20 were p-info and 118 singleton sites. The 340-aa-long COXI protein alignment identified eight variable amino acid positions. Sequence alignment comparisons with *S. mansoni* haplotypes/isolates available in GenBank evidenced only two haplotypes, Sm-*cox*1-H11 and Sm-*cox*1-H16, both found in the Guinean patient, which proved to be identical to two *S. mansoni* sequences previously reported from Côte d'Ivoire (Figure 3).

### 3.4. *cox*1 Haplotype Networks

**Figure 4:**
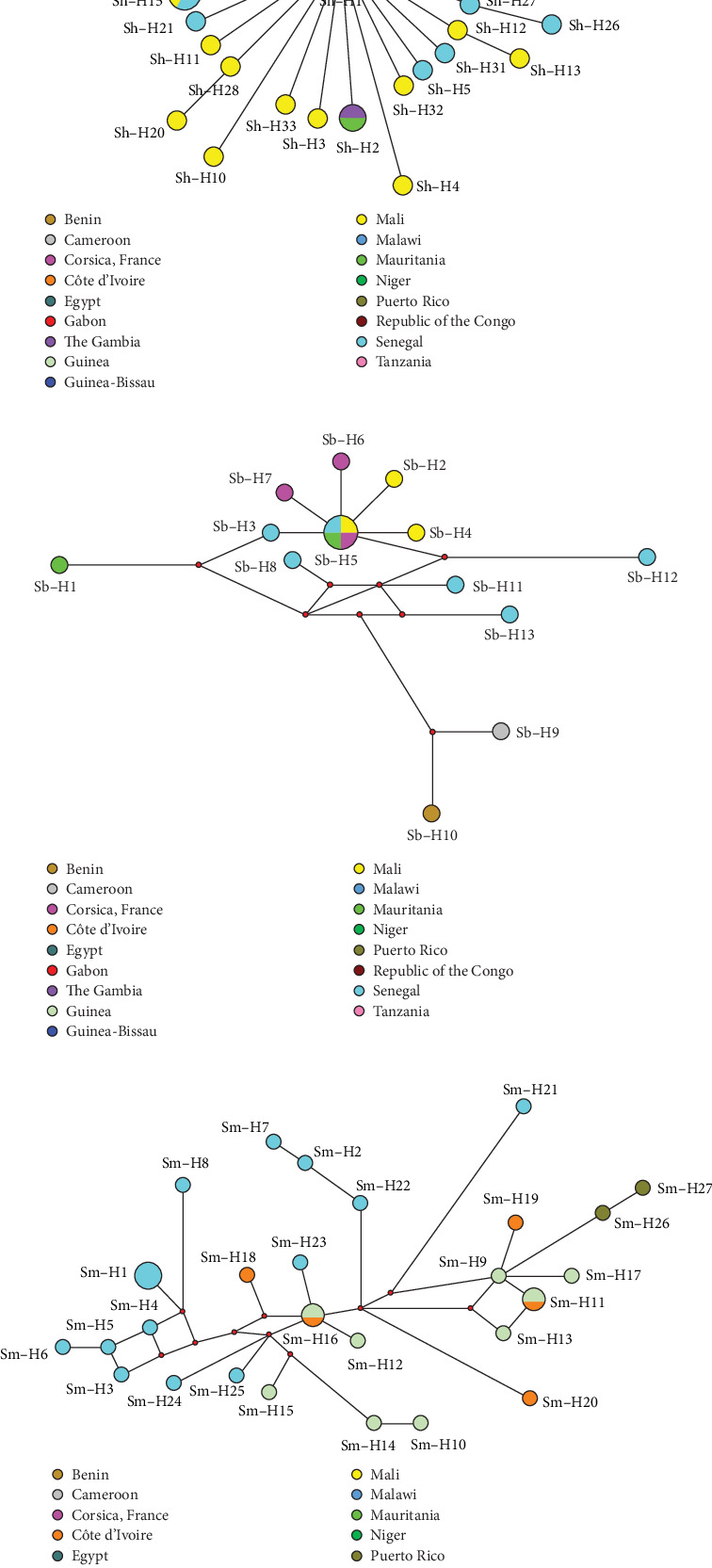
Phylogenetic network based on mtDNA *cox*1 sequences of haplotypes detected in migrant people in Spain according to their country of origin and other southwestern Europe and African and American samples from GenBank: (A) *S. haematobium* (Sh); (B) *S. bovis* (Sb); and (C) *S. mansoni* (Sm). Small red-filled circle represents intermediate haplotype not present in the sample. Mutational steps between haplotypes are represented by a line. The colors represented in the legend correspond with those represented by the haplotype network. Haplotype (H) information detailed in Table S4.

A first network analysis was performed including 42 sequences of *S. haematobium* representing our 34 haplotypes (Sh-*cox*1-H1 to Sh-*cox*1-H34), plus nine sequences representing haplotypes/isolates from Benin, Egypt, Gabon, Malawi, Mali, Niger, Senegal, Republic of the Congo, and Tanzania (Table S4). Sequences were collapsed to 37 haplotypes with a global haplotype diversity (Hd) of 0.9721 showing a star topology radial distribution with a numerical dominant central haplotype, Sh-*cox*1-H1, shared by eight countries. Of the remaining haplotypes, only five of them are shared between two countries: H2 (Gambia and Mauritania), H9 (Guinea Bissau and Mali H9), H14 (Senegal and Benin), H15 (Mali and Senegal), and H22 (Mali and Egypt) (Figure 4A).

The *S. bovis* network was constructed including 16 sequences representing our five haplotypes (Sb-*cox*1-H1 to Sb-*cox*1-H5) plus haplotypes/isolates from Corsica (France), Cameroon, Benin, and Senegal (*cox*1-H6 to H13). The network collapsed the 16 sequences into 13 haplotypes, showing a Hd of 0.9500. The haplotype Sb-*cox*1-H5 detected in our migrant patients from Mali, Mauritania, and Senegal has also been reported in humans in Corsica. This core haplotype is linked with other haplotypes from Corsica as well as with Mali and Senegal. Cameroon and Benin show the most distant haplotypes in the network (Figure 4B).

The third network analysis was performed including 31 sequences of *S. mansoni* representing our 20 haplotypes (Sm-*cox*1-H1 to Sm-*cox*1-H20), plus nine sequences representing haplotypes/isolates from Puerto Rico, Senegal, and Côte d'Ivoire (haplotypes Sm-*cox*1-H21 to Sm-*cox*1-H27) (Table S4). This *cox*1 network was collapsed into 27 different haplotypes with a Hd of 0.9892. Only the haplotypes Sm-*cox*1-H11 and Sm-*cox*1-H16 were shared between Guinea and Côte d'Ivoire. The haplotypes from Senegal appear grouped together but linked to the haplotypes from Guinea and Côte d'Ivoire, which are also closely related together. The Puerto Rican haplotypes appear distant from the African haplotypes (Figure 4C).

### 3.5. *cox*1 Phylogenies

**Figure 5:**
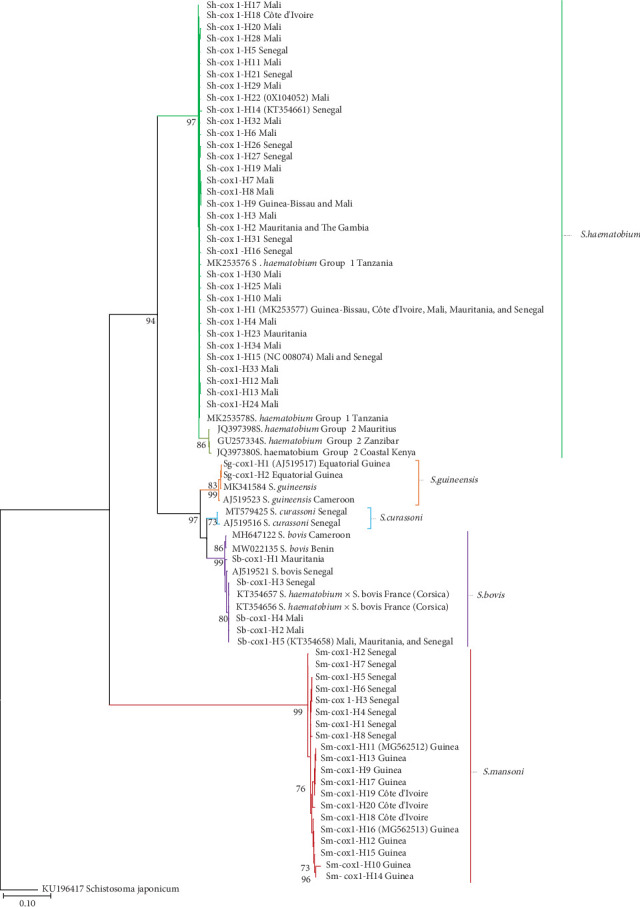
Phylogenetic tree of mtDNA *cox*1 *Schistosoma* species based on maximum likelihood model constructed with 61 (34 *Schistosoma haematobium*, 2 *S. guineensis*, 5 *S. bovis*, and 20 *S. mansoni*) haplotypes from the present study and 22 sequences from the GenBank database (accession numbers are shown in the tree). Tree rooted using the *S. japonicum* sequence (KU196417) as outgroup. The tree is drawn to scale, with branch lengths measured as the number of substitutions per site.

The ML model best fitting our *cox*1 dataset was the Hasegawa–Kishino–Yano model with discrete gamma distribution (HKY + G). The resulting ML tree (log likelihood = −4330.17) was inferred with +G, parameter = 0.1911, and the rate variation model allowed for some sites to be evolutionarily invariant (Figure 5). This analysis involved 84 nucleotide sequences, including *S. haematobium*, *S. bovis*, *S. curassoni*, *S. guineensis*, and *S. mansoni cox*1 haplotypes, other *Schistosoma* isolates or haplotypes from GenBank, and *S. japonicum* (KU196417) as outgroup. There were a total of 1024 positions in the final dataset.

In the ML tree, the 34 *cox*1 haplotypes of *S. haematobium* form a monophyletic clade together with haplotypes representing *S. haematobium* Group 1, which includes parasites from continental Africa. This clade is closely related and well supported (97%) with the clade including *S. haematobium* from Mauritius, Zanzibar, and coastal Kenya, representing *cox*1 haplotypes of *S. haematobium* of Group 2. Thus, all haplotypes detected in migrants in Spain arriving from Mali, Mauritania, The Gambia, Senegal, Côte d'Ivoire, and Guinea-Bissau grouped together in the monophyletic clade corresponding to *S. haematobium* Group 1.

The haplotypes corresponding to *S. guineensis*, *S. curassoni*, and *S. bovis* cluster together in a well-supported (97%) monophyletic clade that includes a monophyletic branch for each species.

Within the *S. bovis* branch, four *S. bovis cox*1 haplotypes detected in migrants in Spain arriving from Mali, Mauritania, and Senegal cluster together with *S. bovis* haplotypes from Europe (France: Corsica) with an 80% of support. The two *S. guineensis cox*1 haplotypes from Equatorial Guinea cluster inside a monophyletic branch of *S. guineensis* that includes other *S. guineensis cox*1 haplotypes from Cameroon.

A basal clade of the tree includes *S. mansoni* haplotypes showing high support (99%). Although clustered in the same clade, the Senegalese *S. mansoni cox*1 haplotypes detected in migrants in Spain are grouped in the same branch, appearing as a sister branch of all haplotypes from Guinea and Côte d'Ivoire.

## 4. Discussion

Molecular techniques combining the *cox*1 gene and nuclear ITSs are commonly used to distinguish *Schistosoma* species and identify hybrids in patients diagnosed in endemic countries [2, 42, 50, 53]. However, such genetic studies are very scarce in patients diagnosed in Europe. They have only been performed in specific cases, such as migrants diagnosed with schistosomiasis with ectopic egg shedding [54, 55] or travelers returning from endemic countries and shedding *Schistosoma* eggs with unusual morphology [56]. To our knowledge, this is the first study based on the combination of nuclear and mitochondrial DNA markers in sub-Saharan migrants diagnosed with schistosomiasis and residing in Spain.

Among the 58 patients included in the study, the prevalence of urogenital schistosomiasis was 91.38% (53/58), while intestinal schistosomiasis was observed in 8.62% (5/58) of cases. Additionally, a single case of coinfection (1.72%) involving both intestinal and urogenital schistosomiasis was detected. There was only one case of a woman among the 58 patients who presented intestinal schistosomiasis due to *Schistosoma guineensis*. After administering the usual treatment with praziquantel 40 mg/kg 1 day, the patient was followed up at 3, 6, and 12 months without evidence of treatment failure. In a clinical practice at the TMUs, the follow-up of patients with schistosomiasis is the same regardless of sex. The only difference is that women with genitourinary schistosomiasis are referred for gynecological assessment to rule out genital involvement.

Our study revealed that 22 out of 54 (40.74%) migrant patients diagnosed with urogenital schistosomiasis (including the single patient with co-infection) were simultaneously shedding pure and hybrid eggs in their urine samples. This simultaneous detection of both pure and hybrid genetic profiles in the same patient is in line with observations made in patients from endemic countries such as Senegal and Côte d'Ivoire [2, 42, 57]. Our five patients diagnosed with intestinal schistosomiasis were carriers of pure infections (*S. mansoni* or *S. guinennesis*).

The presence of *S. haematobium* × *S. bovis* hybrid eggs was confirmed in 15 patients (68.18%) from Mali, Mauritania, and Senegal, out of the 22 patients shedding hybrid eggs. *S*. *haematobium* × *S. bovis* hybrids are the most prevalent and extensively studied in West Africa with human reports in Benin [50], Cameroon [8], Côte d'Ivoire [42], Mali [41], Nigeria [7], and Senegal [2, 53, 58].

We identified *S. haematobium* × *S. curassoni* hybrids in seven patients (31.82%) (including the Senegalese patient in which both *S. haematobium* × *S. bovis* and *S. haematobium* × *S. curassoni* hybrid eggs were observed), from Côte d'Ivoire, Mali, Mauritania, and Senegal, out of the 22 patients with urogenital schistosomiasis shedding hybrid eggs. The existence of *S. haematobium* × *S. curassoni* hybrids has also been reported in Senegal [2] and Mali [41]. Here, we report new human cases of such hybrids for Côte d'Ivoire and Mauritania, so that the interaction between these two species also occurs in more West African countries.

Worth mentioning is the detection of three-species hybrid eggs in a single patient from Mali (Tables 3 and 4), in which *S. bovis* was detected in *cox*1 and *S. haematobium* × *S. curassoni* profile in ITS-2 and 18S, illustrating the interaction between *S. haematobium*, *S. bovis*, and *S. curassoni*. Such three-species hybrids have been previously reported in Niger [59] and Mali [41]. The genetic profile of these three-species hybrid eggs observed in our study supports the hypothesis that they are not first-generation hybrids but the result of parental backcrosses and/or relatively recent hybrid lineages [41, 59].

The low frequency of *S. haematobium* × *S. curassoni* hybrids reported in the literature compared to hybrids resulting from *S. haematobium* and *S. bovis*, as well as the recently identified three-species hybrids, has been attributed to the rarity of these interactions due to the limited distribution of *S. curassoni*, which is found in restricted foci in some West African countries, mainly in Senegal [60]. However, such hybrids may be underestimated because the ITS-2 marker used in most field studies (and also RD-PCR) does not discriminate between *S. bovis* and *S. curassoni*, requiring the use of another nuclear marker, such as the 18S rRNA gene [41, 59], to discriminate [51].

Concerning the genetic signatures using *cox*1 and ITS-2/18S markers together, in the 642 eggs from patients with urinary schistosomiasis, we describe a total of six mito-nuclear signatures, among which only one corresponds to pure *S. haematobium* (83.30%) and five (16.67%) to *S. haematobium* × *S. bovis*, *S. haematobium* × *S. curassoni*, and *S. haematobium* × *S. bovis* × *S. curassoni* hybrid combinations. Of the 60 eggs from patients with intestinal schistosomiasis, two mito-nuclear signatures were obtained corresponding to the pure profiles of *SmxSmSm* (85%) and *SgxSgSg* (15%).

All possible mito-nuclear combinations of *S. haematobium* × *S. bovis* have been detected in eggs collected from patients with urogenital schistosomiasis in sub-Saharan endemic countries [2, 7, 8, 42]. Hybrids, in which *cox*1 shows a *S. bovis* profile and ITSs exhibits *S. haematobium* alleles (*SbxShSh*), have proven to be the most common mito-nuclear signature reported, being detected in Benin [50], Cameroon [8], Côte d'Ivoire [42], Nigeria [7], Senegal [2], and even in France [3]. This mito-nuclear signature proves the mitochondrial introgressive hybridization of *S. haematobium cox*1 by *S. bovis* [3]. Our results agree with previous studies, because we have identified three hybrid mito-nuclear signatures, with *SbxShSh* being the most abundant and detected in 13 patients (nine from Mali, one from Mauritania, and three from Senegal).

No hybrid mito-nuclear signature was detected in stool samples from those patients. Although some studies have reported the presence of miracidia with a pure *S. bovis* signature (*SbxSbSb*) in patients' urine [7, 12, 42], we did not observe such cases in our patients. A few *ShxSm* crosses have also been detected [55, 61].

The infection with *S. guineensis* was confirmed in the patient from Equatorial Guinea who excreted terminal-spined eggs in the stool. This species is restricted to countries of the region of the Guinea Gulf [62]. Interestingly, the possible involvement of *B. truncatus* in its transmission has recently been suggested in Gabon [63], which, if confirmed, could represent a risk of autochthonous transmission of this species in Spain, where *B. truncatus* is present. *S. mansoni* was identified in the lateral-spined eggs collected from the stools of the patients from Côte d'Ivoire, Guinea, and Senegal. It should be noted that the patient from Senegal was also diagnosed with urogenital schistosomiasis and excreted pure *S. haematobium* eggs in his urine, indicating coinfection. *S. mansoni* is widely distributed across the African continent, with both Guinea and Senegal being endemic countries [64]. Furthermore, in Senegal, due to the overlap between the two main species, *S. haematobium* and *S. mansoni*, there are increasing reports of mixed and hybrid infections in the northern region of the country in recent years [65]. A priori, *S. mansoni* would not pose a risk of autochthonous transmission in Spain due to the absence of a competent vector, freshwater snails of the genus *Biomphalaria*.

In Spain, as in the rest of Europe, the main profile of sub-Saharan migrants diagnosed with schistosomiasis corresponds to young men mainly from West Africa, with Mali and Senegal standing out as countries of origin [66, 67]. Given the high prevalence of hybrids between human species, such as *S. haematobium*, and animal species, such as *S. bovis* and *S. curassoni*, in their origin countries, it could be expected that these types of hybrids would be observed in migrant patients in Europe, as demonstrated in our study.

Although crosses between *S. haematobium* and *S. bovis* or *S. haematobium* and *S. curassoni* are thought to generate lineages with increased virulence compared to the parental species [51], very little is known about the impact of these hybrids on pathology, morbidity, and treatment response. Recently, a study from Senegal observed a different morbidity profile in hybrid *S. haematobium* × *S. bovis* infections compared to *S. haematobium* infections. Individuals infected with *S. haematobium* × *S. bovis* hybrids showed an increase in hepatic morbidity and a decrease in urogenital morbidity, as well as a reduction in improvement after treatment with praziquantel, compared with patients without hybrids [68]. Similarly, in migrant patients in Spain coming from West African countries, the presence of *Schistosoma* hybrids seems to cause increased morbidity in infected individuals. However, it does not appear to result in differences in diagnostic tests or in clinical and analytical responses after treatment [69]. These data may be similar for hybrid infections of *S. haematobium* × *S. curassoni*, as the latter is also an animal species that causes intestinal schistosomiasis.

The significant percentage of migrant patients with urogenital schistosomiasis carrying hybrids (40.74%), especially those from Mali (13 out of 24 patients, 54.16%), followed by Guinea-Bissau (1/3, 33,33%), Mauritania (3/11, 27,27%), and Senegal (4/17, 23.59%), highlights the importance of monitoring and detecting these hybrid infections, as well as the different types of hybrids involved, to understand their impact on morbidity and treatment response.

Molecular haplotyping by sequencing has allowed us to describe 12 ribosomal and 61 mitochondrial haplotypes among migrant patients diagnosed with schistosomiasis in Spain. The ITS1-5.8S-ITS2 region revealed seven pure haplotypes: five of *S. haematobium*, one of *S. guineensis*, and one of *S. mansoni* and five hybrid haplotypes of *S. haematobium* × *S. bovis* and *S. haematobium* × *S. curassoni* (Figure 2). The *cox*1 sequencing detected 34 pure haplotypes corresponding to *S. haematobium*; five to *S. bovis*; two to *S. guineensis*; and 20 to *S. mansoni*. As expected [60], a low level of genetic structuration of *S. haematobium* appears when compared to *S. bovis* or *S. mansoni* (Figure 4). All of these here-described haplotypes are first findings in Spain-imported schistosomes and new for the countries studied, with the exception of the ribosomal haplotypes, which were all previously reported in other African countries [50, 70, 71] (Figure 2 and Table S2) or the scarce eight mitochondrial *cox*1 haplotypes of the total of the here-described 61, which showed 100% sequence identity with other isolates or haplotypes previously described [3, 55, 72–75] (Figure 3 and Table S3).

Across the sub-Saharan African region, *S. haematobium cox*1 was confirmed to cluster into two groups: Group 1 including parasites from mainland Africa and Group 2 including parasites from Indian Ocean islands and neighboring coastal regions of Africa [76]. The *cox*1 phylogenetic tree grouped our 34 *S. haematobium* haplotypes into Group 1, as expected based on the country of origin of our patients (Figure 5). The phylogenetic relationship between *S. bovis cox*1 haplotypes in patients from Mali, Mauritania, and Senegal and haplotypes from Europe (France: Corsica) clearly supports the origin of Corsican hybrids attributed to Senegal [12] but also suggest that it is difficult to establish a single country as origin of these hybrids found in Corsica. Indeed, a recent study has suggested that the origin of these hybrids could also be attributed to Benin, based on an 881-bp-partial *cox*1 phylogenetic tree [50]. The high number of *cox*1 haplotypes of *S. mansoni* entering with patients diagnosed with intestinal schistosomiasis in Spain is worth mentioning. This confirms the great genetic variability of this species in its countries of origin and illustrates the *S. mansoni* diversity, as previously reported in children in other countries as Yemen [77].

## 5. Conclusion

Our molecular haplotyping and phylogenetic analysis of the different genetic profiles of *Schistosoma* (pure and hybrid) species entering in Spain, and consequently in Europe, pronouncedly contributes to (i) demonstrate the great genetic variability of schistosome strains arriving to a nonendemic country; (ii) correlate the geographical origin of the patients with the different types of pure species and/or hybrids; (iii) allow to detect the presence of hybrids “at distance,” as indeed we have identified hybrids in countries (Guinea-Bissau and Mauritania) lacking such information; (iv) allow to henceforth assess the correlation between different haplotypes from migrant patients and their respective pathologies, clinics, and treatment; and (v) identify the pure and/or hybrid species that are entering Spain and warn about possible sources of autochthonous transmission.

## Figures and Tables

**Table 1:** Number of *Schistosoma* spp. eggs analyzed according to the country of origin of the migrant patients and the type of sample from which they were recovered.

Country of origin	Number of patients	Type of sample	Terminal-spined eggs	Lateral-spined eggs
Guinea-Bissau	3	Urine	36	—
Côte d'Ivoire	1	Urine	13	—
Mali	24	Urine	283	—
Mauritania	8	Urine	133	—
Senegal	16	Urine	201	—
Senegal	1	Urine and stool	16	—
The Gambia	1	Urine	16	—
Guinea	1	Stool	—	12
Equatorial Guinea	1	Stool	10	—
Senegal	1	Urine and stool	—	23
Senegal	1	Stool	—	8
Côte d'Ivoire	1	Stool	—	8
Total	58	—	708	51

**Table 2:** Total number of eggs processed for the creation of a mito-nuclear signature and haplotype identification, along with the markers and techniques used for each purpose.

	Mito-nuclear signature							Haplotype identification					
Genetic markers	*cox*1 RD-PCR			ITS-2 sequencing (313 bp)			18S sequencing (1369 bp)	ITS-1, 5.8S, ITS-2 sequencing (927 bp)			*cox*1 sequencing (1024/1052 bp)		
Type of sample	Urine	Stool		Urine	Stool		Urine	Urine	Stool		Urine	Stool	
Egg morphology	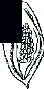	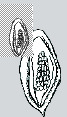	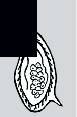	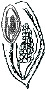	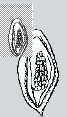	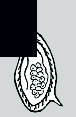	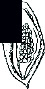	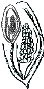	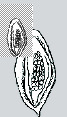	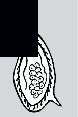	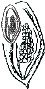	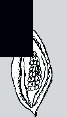	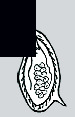
Processed eggs	698	10	51	698	10	51	30	251	10	24	251	10	24
Total processed eggs	**759**			**759**			**30**	**285**			**285**		

*Note*: The bold values are to highlight the total number of eggs processed with each technique, making it easier to follow the results in the text.

Abbreviation: bp, base pairs.

**Table 3:** Genetic profile based on both *cox*1 RD-PCR and ITS-2/18S sequence analysis of eggs successfully genotyped (*n*) by country of origin and type of sample (urine or stool).

Genotyped eggs	Country	(*n*)	RD-PCR			Sequence	Mito-nuclear signature	Genetic profile	Classification
			*Sh cox1*	*Sb* or *Sc cox1*	*Sm cox1*	ITS-2			
Eggs from urine *n* = 642	The Gambia	15	15	—	—	*ShSh*	*ShxShSh*	*S. haematobium*	Pure
	Guinea-Bissau	36	35	—	—	*ShSh*	*ShxShSh*	*S. haematobium*	Pure
			**1**	—	—	*ShSb/Sc⁣* ^*∗*^	*ShxShSb/Sc⁣* ^*∗*^	*S. haematobium* × *S. bovis/S. curassoni*	Hybrid
	Côte d'Ivoire	12	8	—	—	*ShSh*	*ShxShSh*	*S. haematobium*	Pure
			**4**	—	—	*ShSc*	*ShxShSc*	*S. haematobium* × *S. curassoni*	Hybrid
	Mali	249	195	—	—	*ShSh*	*ShxShSh*	*S. haematobium*	Pure
		—	—	40	—	*ShSh*	*SbxShSh*	*S. haematobium* × *S. bovis*	Hybrid
		—	—	**5**	—	*ShSc*	*SbxShSc*	*S. haematobium* × *S. bovis* × *S. curassoni*	Hybrid
		—	**1**	—	—	*ShSb/Sc⁣* ^*∗*^	*ShxShSb/Sc⁣* ^*∗*^	*S. haematobium* × *S. bovis/S. curassoni*	Hybrid
		—	**5**	—	—	*ShSc*	*ShxShSc*	*S. haematobium* × *S. curassoni*	Hybrid
		—	**3**	—	—	*ShSb*	*ShxShSb*	*S. haematobium* × *S. bovis*	Hybrid
	Mauritania	128	109	—	—	*ShSh*	*ShxShSh*	*S. haematobium*	Pure
		—	—	10	—	*ShSh*	*SbxShSh*	*S. haematobium* × *S. bovis*	Hybrid
		—	—	**2**	—	*ShSb*	*SbxShSb*	*S. haematobium* × *S. bovis*	Hybrid
		—	**7**	—	—	*ShSc*	*ShxShSc*	*S. haematobium* × *S. curassoni*	Hybrid
	Senegal	202	173	—	—	*ShSh*	*ShxShSh*	*S. haematobium*	Pure
		—	—	27	—	*ShSh*	*SbxShSh*	*S. haematobium* × *S. bovis*	Hybrid
		—	**2**	—	—	*ShSc*	*ShxShSc*	*S. haematobium* × *S. curassoni*	Hybrid

Eggs from stool *n* = 60	Guinea	12	—	—	12	*SmSm*	*SmxSmSm*	*S. mansoni*	Pure
	Equatorial Guinea	9	—	—	—	*SgSg*	*SgxSgSg⁣* ^*∗∗*^	*S. guineensis*	Pure
	Senegal	31	—	—	31	*SmSm*	*SmxSmSm*	*S. mansoni*	Pure
	Côte d'Ivoire	8	—	—	8	*SmSm*	*SmxSmSm*	*S. mansoni*	Pure

*Note:* In bold = eggs with double chromatogram peaks at the polymorphic positions that discriminate between *S. haematobium* and *S. bovis/S. curassoni* in their ITS-2 sequences; in this case, 18S sequences were used for species identification.

Abbreviations: *Sb*, *S. bovis*; *Sc*, *S. curassoni*; *Sg*, *S. guineensis; Sh*, *Schistosoma haematobium*; *Sm*, *S. mansoni*.

*⁣* ^*∗*^Indeterminate due to the lack of DNA for 18S rRNA gene sequencing.

*⁣* ^*∗∗*^Mito-nuclear signature performed on the information obtained from mtDNA *cox*1 sequencing.

**Table 4:** Number of patients' carriers of pure or hybrid infections according the different types of mito-nuclear signatures detected in eggs collected from their urine or stool.

Number of patients	Country	Genetic profiles		
		Pure	Hybrid	
Urine samples				
1	The Gambia	*ShxShSh*	—	—
2	Guinea Bissau	*ShxShSh*	—	—
1	Guinea Bissau	*ShxShSh*	*ShxShSb/Sc⁣* ^*∗*^	—
1	Côte d'Ivoire	*ShxShSh*	*ShxShSc*	—
11	Mali	*ShxShSh*	—	—
8	Mali	*ShxShSh*	*SbxShSh*	—
1	Mali	*ShxShSh*	*SbxShSc*	—
1	Mali	*ShxShSh*	*ShxShSb/Sc⁣* ^*∗*^	*SbxShSh*
2	Mali	*ShxShSh*	*ShxShSc*	—
1	Mali	*ShxShSh*	*ShxShSb*	—
5	Mauritania	*ShxShSh*	—	—
2	Mauritania	*ShxShSh*	*ShxShSc*	—
1	Mauritania	*ShxShSh*	*SbxShSh*	*SbxShSb*
13	Senegal	*ShxShSh*	—	—
2	Senegal	*ShxShSh*	*SbxShSh*	—
1	Senegal	*ShxShSh*	*SbxShSh*	*ShxShSc*
1	Senegal	*ShxShSh*	*ShxShSc*	—
Stool samples				
1	Guinea	*SmxSmSm*	—	—
1	Equatorial Guinea	*SgxSgSg*	—	—
2	Senegal	*SmxSmSm*	—	—
1	Côte d'Ivoire	*SmxSmSm*	—	—

*⁣* ^*∗*^Indeterminate due to the lack of DNA for 18S rRNA gene sequencing.

## Data Availability

The data that support the findings of this study are openly available in the GenBank database at https://www.ncbi.nlm.nih.gov/genbank, under accession numbers PP905237-PP905248 and PP905315-PP905375.

## Supporting Information

Supporting Information Contains Tables S1–S4, and the table captions are listed as follows: Table S1: Species-specific variable positions in the ITS-1, ITS-2, and 18S rDNA sequences used to discriminate the three *Schistosoma* species and hybrids (when alleles of both species in combination were detected in their sequence chromatogram). Table S2: Distribution of the 85 nuclear ribosomal DNA sequences including the complete intergenic region (ITS1-5.8S-ITS2) from GenBank used for comparison purposes, according to their hosts and geographical origin: (A) 59 sequences of *S. haematobium*, *S. curassoni*, *S. guineensis*, *S. bovis*, and *S. haematobium* × *S. bovis* and the corresponding 21 haplotypes they provided; (B) 27 sequences of *S. mansoni* and the corresponding four haplotypes they provided. Bp = base pairs of the ITS rDNA region available in GenBank for each sample. Identical sequences of different haplotype/isolate are grouped and separated by horizontal lines. Table S3: Distribution of the 77 *cox*1 sequences from GenBank used for comparison purposes, according to their hosts and geographical origin: (A) 19 sequences of *S. haematobium* and the corresponding nine haplotypes they provided; (B) 48 sequences of *S. bovis* and the corresponding 48 haplotypes they provided; (C) four sequences of *S. guineensis* and the corresponding three haplotypes they provided; (D) six sequences of *S. mansoni* and the corresponding five haplotypes they provided. Bp = base pairs of the *cox*1 gene available in GenBank for each sample. Identical sequences of different haplotype/isolate are grouped and separated by horizontal lines. Table S4: Distribution of the *cox*1 sequences analyzed for haplotype networks of (A) *S. haematobium* (*Sh*); (B) *S. bovis* (*Sb*); and (C) *S. mansoni* (*Sm*) and the corresponding haplotypes they provided, according to their country and host. H, haplotype.
