# Supplementary material for: Imported Schistosomiasis in Southwestern Europe: Wide Variation of Pure and Hybrid Genotypes Infecting Sub-Saharan Migrants
Source: Transbound Emerg Dis. 2025 Apr 18;2025:6614509. doi: 10.1155/tbed/6614509 (PMC12298521; doi:10.1155/tbed/6614509)
Supplement: Supporting Information — Contains Tables S1–S4, and the table captions are listed as follows: Table S1: Species-specific variable positions in the ITS-1, ITS-2, and 18S rDNA sequences used to discriminate the three Schistosoma species and hybrids (when alleles of both species in combination were detected in their sequence chromatogram). Table S2: Distribution of the 85 nuclear ribosomal DNA sequences including the complete intergenic region (ITS1-5.8S-ITS2) from GenBank used for comparison purposes, according to their hosts and geographical origin: (A) 59 sequences of S. haematobium, S. curassoni, S. guineensis, S. bovis, and S. haematobium × S. bovis and the corresponding 21 haplotypes they provided; (B) 27 sequences of S. mansoni and the corresponding four haplotypes they provided. Bp = base pairs of the ITS rDNA region available in GenBank for each sample. Identical sequences of different haplotype/isolate are grouped and separated by horizontal lines. Table S3: Distribution of the 77 cox1 sequences from GenBank used for comparison purposes, according to their hosts and geographical origin: (A) 19 sequences of S. haematobium and the corresponding nine haplotypes they provided; (B) 48 sequences of S. bovis and the corresponding 48 haplotypes they provided; (C) four sequences of S. guineensis and the corresponding three haplotypes they provided; (D) six sequences of S. mansoni and the corresponding five haplotypes they provided. Bp = base pairs of the cox1 gene available in GenBank for each sample. Identical sequences of different haplotype/isolate are grouped and separated by horizontal lines. Table S4: Distribution of the cox1 sequences analyzed for haplotype networks of (A) S. haematobium (Sh); (B) S. bovis (Sb); and (C) S. mansoni (Sm) and the corresponding haplotypes they provided, according to their country and host. H, haplotype. [file 6614509.f1.doc]

**SUPPLEMENTARY TABLES TO:**

De Elias-Escribano A, Artigas P, Salas-Coronas J, Luzon-Garcia MP, Reguera-Gomez M, Sanchez-Marques R, Salvador F, Boissier J, Mas-Coma S, Bargues MD. Imported schistosomiasis in southwestern Europe: wide variation of pure and hybrid genotypes infecting sub-Saharan migrants. *Transboundary and Emerging Diseases. 2024.*

Supplementary TABLE S1: Species-specific variable positions in the ITS-1, ITS-2 and 18S rDNA sequences used to discriminate the three *Schistosoma* species and hybrids (when alleles of both species in combination were detected in their sequence chromatogram).

| *Schistosoma* spp. | rDNA marker | | | | | | | | | |
| --- | --- | --- | --- | --- | --- | --- | --- | --- | --- | --- |
|  | ITS-1 | ITS-2 | | | | 18S | | | | |
|  | 51 | 703 | 758 | 808 | 878 | 225 | 250 | 297 | 685 | 1184 |
| *S. haematobium* | A | G | C | G | C | T | C | T | T | A/G |
| *S. bovis* | G | A | T | A | T | C | T | C | C | G |
| *S. curassoni* | A | A | T | A | T | T | T | T | T | G |

Supplementary TABLE S2: Distribution of the 85 nuclear ribosomal DNA sequences including the complete intergenic region (ITS-1, 5.8S and ITS-2) from GenBank used for comparison purposes, according to their hosts and geographical origin: **A)** 59 sequences of *S. haematobium, S. curassoni, S. guineensis, S. bovis*, *S. haematobium x S. bovis* and the corresponding 21 haplotypes they provided**; B)** 27 sequences of *S. mansoni*, and the corresponding 4 haplotypes they provided. Bp = base pairs of the ITS rDNA region available in GenBank for each sample. Identical sequences of different haplotype /isolate are grouped and separated by horizontal lines.

A)

| **Haplotype/isolate** | **Organism** | **GenBank**  **Acc.No.** | **Host** | **Locality** | **Country** | **Length**  **(bp)** |
| --- | --- | --- | --- | --- | --- | --- |
| tdSchHaem2.1 | *S. haematobium* | [OX103963](https://www.ncbi.nlm.nih.gov/nucleotide/OX103963.1?report=genbank&log$=nucltop&blast_rank=2&RID=WD17HYG2013) | *-* | - | Tanzania | 927 |
| tdSchHaem1.1 | *S. haematobium* | [OX104046](https://www.ncbi.nlm.nih.gov/nucleotide/OX104046.1?report=genbank&log$=nucltop&blast_rank=3&RID=WD17HYG2013) | *-* | - | Egypt | 927 |
| KE14_26 | *S. haematobium* | MT158873 | *Homo sapiens* | Kessounou | Benin | 924 |
| pSHTSA1- A2, pSHTSC1, pSHTSB3, pSHTSF3 | *S. haematobium* | Z21716 | clone | - | Mali | 923 |
| Mwenje_BGL_7 | *S. haematobium* | MT884914 | *Bulinus globosus* | - | Zimbabwe | 927 |
| DK23_5 | *S. haematobium x S. bovis* | MT158878 | *Homo sapiens* | Kessounou | Benin | 924 |
| - | *S. haematobium* | MG554667 | - | - | Côte d'Ivoire | 927 |
| ITS_2019 | *S. haematobium* | MW130296 | *-* | Corsica | France | 927 |
| KU10_Hs_  Profile8 | *S. haematobium x S. bovis* | MW027655 | *Homo sapiens* | Kessounou | Benin | 927 |
| DK54_16 | *S. haematobium x S. bovis* | MT158876 | *Homo sapiens* | Kessounou | Benin | 924 |
| hyShITSBt | *S. haematobium x S. bovis* | FJ588857 | *Bulinus truncatus* | Senegal River Basin | Senegal | 926 |
| hyShITSmir | *S. haematobium x S. bovis* | FJ588858 | *Homo sapiens* | Senegal River Basin | Senegal | 926 |
| hyShITSBg | *S. haematobium x S. bovis* | FJ588859 | *Bulinus globosus* | Senegal River Basin | Senegal | 926 |
| hymixShITSmir | *S. haematobium x S. bovis* | FJ588860 | *Homo sapiens* | Senegal River Basin | Senegal | 926 |
| ShITSmir | *S. haematobium* | FJ588861 | *Homo sapiens* | Senegal River Basin | Senegal | 926 |
| ShITSb | *S. haematobium* | JQ397401 | - | - | Mali | 926 |
| - | *S. haematobium x S. bovis* | MK358844 | *Homo sapiens* | Nsanje | Malawi | 915 |
| - | *S. haematobium* | MK358856 | *Homo sapiens* | Mangochi | Malawi | 915 |
| - | *S. haematobium* | MK358857 | *Homo sapiens* | Nsanje | Malawi | 915 |
| - | *S. haematobium* | MK358858 | *Homo sapiens* | Chikhawa | Malawi | 915 |
| - | *S. haematobium* | MK797748 | *Rattus rattus* | - | France | 926 |
| BK17_BB7.2 | *S. haematobium* | MT580953 | *Homo sapiens* | Barkedji | Senegal | 926 |
| BK17_LLod1.2 | *S. haematobium* | MT580954 | *Bulinus umbilicatus* | Loumbel Lode | Senegal | 926 |
| BK17_FH21.6 | *S. haematobium x S. bovis* | MT580955 | *Homo sapiens* | Loumbel Lana | Senegal | 926 |
| RT15_Ye1-10.6 | *S. haematobium x S. bovis* | MT580957 | *Bulinus truncatus* | Yetti-Yone, Lac de Guiers | Senegal | 926 |
| RT16_NW51.3 | *S. haematobium* | MT580959 | *Homo sapiens* | Richard Toll | Senegal | 926 |
| - | *S. haematobium* | GU257398 |  | Zanzibar | Tanzania | 926 |
| KU5_Hs_Profile10 | *S. haematobium x S. bovis* | MW027657 | *Homo sapiens* | Kessounou | Benin | 927 |
| DK23_23 | *S. haematobium x S. bovis* | MT158879 | *Homo sapiens* | Kessounou | Benin | 924 |
| BK17_KG1.4 | *S. curassoni* | MT580946 | *Bulinus umbilicatus* | Kangaledji | Senegal | 926 |
| BK17_BKG7.7 | *S. curassoni* | MT580947 | *Capra hircus* | Barkedji | Senegal | 926 |
| BK16.1_  BB8liv.8 | *S. bovis*  *x S. curassoni* | MT580948 | *Bos indicus* | Linguere | Senegal | 926 |
| RT17_MbG8.6 | *S. curassoni* | MT580961 | *Capra hircus* | Mbane, Lac de Guiers | Senegal | 926 |
| V20M2_Rr_  Profile1 | *S. bovis* | MW027648 | *Rattus rattus* | Vekky | Benin | 927 |
| tdSchBovi2.1 | *S. bovis* | OX104095 | - | - | Kenya | 927 |
| tdSchBovi1.1 | *S. bovis* | [OX103954](https://www.ncbi.nlm.nih.gov/nucleotide/OX103954.1?report=genbank&log$=nucltop&blast_rank=3&RID=WD0FHF8C013) | - | - | Spain | 927 |
| tdSchGui1.1 | *S. guineensis* | [OX103898](https://www.ncbi.nlm.nih.gov/nucleotide/OX103898.1?report=genbank&log$=nucltop&blast_rank=4&RID=WD0FHF8C013) | *-* | - | São Tomé and Principe | 927 |
| BK19_1 | *S. bovis* | MT158872 | Cows | Kessounou | Benin | 924 |
| SbITSBt | *S. bovis* | FJ588862 | *Bulinus truncatus* | Upper Senegal River Basin | Senegal | 926 |
| BK16.3_3BB13st.1 | *S. bovis* | MT580950 | Cattle, goat and sheep | Linguere | Senegal | 926 |
| RT17_DB19.8 | *S. bovis* | MT580958 | *Bos indicus* | Didjiery | Senegal | 926 |
| V20M1_Rr_  Profile2 | *S. bovis* | MW027649 | *Rattus rattus* | Vekky | Benin | 927 |
| BK19_2 | *S. haematobium x S. bovis* | MT158874 | Cows | Kessounou | Benin | 924 |
| V20M8_Rr_Profile4 | *S. bovis* | MW027651 | *Rattus rattus* | Vekky | Benin | 927 |
| V132M1_Rr_Profile3 | *S. bovis* | MW027650 | *Rattus rattus* | Vekky | Benin | 927 |
| K14M2_Mn_Profile5 | *S. haematobium x S. bovis* | MW027652 | *Homo sapiens, M. natalensis* | Kessounou | Benin | 927 |
| BK24_4 | *S. haematobium x S. bovis* | MT158881 | *Homo sapiens,* Cows | Kessounou | Benin | 924 |
| BK24_1 | *S. haematobium x S. bovis* | MT158875 | Cows | Kessounou | Benin | 924 |
| K14M1_Mn_Profile6 | *S. haematobium x S. bovis* | MW027653 | *Homo sapiens* | Kessounou | Benin | 926 |
| BK16_LLA15.7 | *S. haematobium x S. bovis* | MT580951 | *Homo sapiens* | Loumbel Lana | Senegal | 926 |
| BK16_LL1.1 | *S. haematobium x S. bovis* | MT580952 | *Homo sapiens* | Loumbel Lana | Senegal | 926 |
| RT16_NG55.5 | *S. haematobium x S. bovis* | MT580960 | *Homo sapiens* | Richard Toll | Senegal | 926 |
| KU9_Hs_Profile7 | *S. haematobium x S. bovis* | MW027654 | *Homo sapiens* | Kessounou | Benin | 927 |
| DK23_21 | *S. haematobium x S. bovis* | MT158877 | *Homo sapiens* | Kessounou | Benin | 924 |
| KU1_Hs_Profile11 | *S. haematobium x S. bovis* | MW027658 | *Homo sapiens* | Kessounou | Benin | 927 |
| DK23_11 | *S. haematobium x S. bovis* | MT158880 | *Homo sapiens* | Kessounou | Benin | 924 |
| RT16.1_Th1.2 | *S. haematobium x S. bovis* | MT580956 | *Bulinus truncatus* | Thiago, Lac de Guiers | Senegal | 926 |
| KU3_Hs_Profile9 | *S. haematobium x S. bovis* | MW027656 | *Homo sapiens* | Kessounou | Benin | 927 |
| Kg2_1 | *S. haematobium x S. bovis* | MT158882 | *Homo sapiens,* Cows | Kessounou | Benin | 924 |

B)

| **Haplotype/isolate** | **Organism** | **GenBank**  **Acc.No.** | **Host** | **Locality** | **Country** | **Length**  **(bp)** |
| --- | --- | --- | --- | --- | --- | --- |
| - | *S. mansoni* | AF531314 | *Biomphalaria sudanica* | Lake Victoria | Tanzania | 927 |
| "SmITSLALV" | *S. mansoni* | FJ750523 | *Homo sapiens* | Lake Albert región | Uganda | 923 |
| ITSa1 | *S. mansoni* | JQ289742 | - | - | Brazil | 915 |
| ITSa2 | *S. mansoni* | JQ289743 | - | - | Senegal | 915 |
| ITSa3 | *S. mansoni* | JQ289744 | - | - | Mali | 915 |
| ITSa4 | *S. mansoni* | JQ289745 | - | - | Nigeria | 915 |
| ITSa5 | *S. mansoni* | JQ289746 | - | - | Uganda | 915 |
| ITSa6 | *S. mansoni* | JQ289747 | - | - | Kenya | 915 |
| ITSa7 | *S. mansoni* | JQ289748 | - | - | Saudi Arabia | 915 |
| ITSa8 | *S. mansoni* | JQ289749 | - | - | Oman | 915 |
| ITSa9 | *S. mansoni* | JQ289750 | - | - | Egypt | 915 |
| "X2" | *S. mansoni* | MF776590 | *Mastomys huberti* | Nder, Lac de Guiers | Senegal | 903 |
| JE191 | *S. mansoni* | AY446082 | *Homo sapiens* | Kariba Dam | Zambia | 927 |
| ITSb1 | *S. mansoni* | JQ289751 | - | - | Brazil | 915 |
| ITSb2 | *S. mansoni* | JQ289752 | - | - | Senegal | 915 |
| ITSb3 | *S. mansoni* | JQ289753 | - | - | Uganda | 915 |
| ITSb4 | *S. mansoni* | JQ289754 | - | - | Kenya | 915 |
| JE21 | *S. mansoni* | AY446079 | *Homo sapiens* | Sumidouro | Brazil | 927 |
| - | *S. mansoni* | KX011041 | *Gorilla gorilla gorilla* | - | Gabon | 955 |
| - | *S. mansoni* | KX011042 | *Pan troglodytes troglodytes* | - | Gabon | 955 |
| ITSc1 | *S. mansoni* | JQ289755 | - | - | Mali | 915 |
| ITSc2 | *S. mansoni* | JQ289756 | - | - | Kenya | 915 |
| - | *S. mansoni* | MG554659 | - | - | Côte d'Ivoire | 902 |
| JE27 | *S. mansoni* | AY446081 | *Biompahalaria pfeifferi* | Makueni, Makindu Town | Kenya | 927 |
| ITSd1 | *S. mansoni* | JQ289757 | - | - | Senegal | 915 |
| ITSd2 | *S. mansoni* | JQ289758 | - | - | Nigeria | 915 |
| ITSd3 | *S. mansoni* | JQ289759 | - | - | Kenya | 915 |

Supplementary TABLE S3: Distribution of the 77 *cox*1 sequences from GenBank used for comparison purposes, according to their hosts and geographical origin: **A)** 19 sequences of *S. haematobium,* and the corresponding 9 haplotypes they provided; **B)** 48 sequences of *S. bovis,* and the corresponding 48 haplotypes they provided; **C)** 4 sequences of *S. guineensis,* and the corresponding 3 haplotypes they provided; **D)** 6 sequences of *S. mansoni,* and the corresponding 5 haplotypes they provided. Bp = base pairs of the *cox*1 gene available in GenBank for each sample. Identical sequences of different haplotype/isolate are grouped and separated by horizontal lines.

A)

| **Haplotype/**  **isolate** | **Organism** | **GenBank**  **Acc.No.** | **Host** | **Locality** | **Country** | **Length**  **(bp)** |
| --- | --- | --- | --- | --- | --- | --- |
| G1 | *S. haematobium* | MK253577 | *Homo sapiens* | Mwera | Tanzania | 1806 |
| Pointe  Noire | *S. haematobium* | KY967520 | *Homo sapiens* | Pointe Noire | Republic of the Congo | 1175 |
| - | *S. haematobium* | KT354659 | *Mesocricetus auratus* | Ekouk | Gabon | 1175 |
| - | *S. haematobium* | KT354660 | *Mesocricetus auratus* | Melen | Gabon | 1175 |
| Malawi-  LgHap2 | *S. haematobium* | EU567128 | *Bulinus globosus* | Lake Malawi-Likoma | Malawi | 1164 |
| G1 | *S. haematobium* | MK253567 | *Homo sapiens* | Daikaina Bell | Niger | 1806 |
| - | *S. haematobium* | KT354661 | *Mesocricetus auratus* | Toho | Benin | 1175 |
| tdSchHaem1.1 | *S. haematobium* | OX104052 | *-* | - | Egypt | 1806 |
| - | *S. haematobium* | MW067222 | *Mesocricetus auratus* | - | Egypt | 1806 |
| - | *S. haematobium* | MW067223 | *Mesocricetus auratus* | - | Egypt | 1806 |
| - | *S. haematobium* | MW067224 | *Mesocricetus auratus* | - | Egypt | 1806 |
| - | *S. haematobium* | MW067225 | *Mesocricetus auratus* | - | Egypt | 1806 |
| - | *S. haematobium* | MW067226 | *Mesocricetus auratus* | - | Egypt | 1806 |
| - | *S. haematobium* | MW067227 | *Mesocricetus auratus* | - | Egypt | 1806 |
| Malawi-LgHap1 | *S. haematobium* | EU567127 | *Bulinus globosus* | Lake Malawi-Likoma | Malawi | 1164 |
| - | *S. haematobium* | NC_008074 | *Mesocricetus auratus* | - | Mali | 1541 |
| 3572 | *S. haematobium* | AJ519520 | - | Mbodiene | Senegal | 1224 |
| G1 | *S. haematobium* | MK253576 | *Homo sapiens* | Kitope | Tanzania | 1806 |
| G1 | *S. haematobium* | MK253578 | *Homo sapiens* | Karma | Niger | 1806 |

B)

| **Haplotype/**  **isolate** | **Organism** | **GenBank**  **Acc.No.** | **Host** | **Locality** | **Country** | **Length**  **(bp)** |
| --- | --- | --- | --- | --- | --- | --- |
| Sh_Corsica1 | *S. haematobium* x *S. bovis* | KT354656 | *Homo sapiens* | Corsica | France | 1174 |
| Sh_Corsica2 | *S. haematobium* x *S. bovis* | KT354657 | *Homo sapiens* | Corsica | France | 1174 |
| Sh_Corsica3 | *S. haematobium* x *S. bovis* | KT354658 | *Homo sapiens* | Corsica | France | 1174 |
| B2 | *S. bovis* | AJ519521 | - | St Louis | Senegal | 1224 |
| Ngaoundere-A1-M13 | *S. bovis* | MH647122 | Cows | - | Cameroon | 1050 |
| Ngaoundere-A1-M11 | *S. bovis* | MH647123 | Cows | - | Cameroon | 1050 |
| Ngaoundere-A1-M12 | *S. bovis* | [MH647124](https://www.ncbi.nlm.nih.gov/nucleotide/MH647124.1?report=genbank&log$=nucltop&blast_rank=27&RID=XF9DE7JX01N) | Cows | - | Cameroon | 1050 |
| Ngaoundere-A1-M14 | *S. bovis* | MH647125 | Cows | - | Cameroon | 1050 |
| Ngaoundere-A1-M18 | *S. bovis* | [MH647127](https://www.ncbi.nlm.nih.gov/nucleotide/MH647127.1?report=genbank&log$=nucltop&blast_rank=26&RID=XF9DE7JX01N) | Cows | - | Cameroon | 1050 |
| Ngaoundere-A1-F19 | *S. bovis* | [MH647128](https://www.ncbi.nlm.nih.gov/nucleotide/MH647128.1?report=genbank&log$=nucltop&blast_rank=35&RID=XF947NTT01N) | Cows | - | Cameroon | 1050 |
| Ngaoundere-A1-F11 | *S. bovis* | [MH647129](https://www.ncbi.nlm.nih.gov/nucleotide/MH647129.1?report=genbank&log$=nucltop&blast_rank=15&RID=XF947NTT01N) | Cows | - | Cameroon | 1050 |
| Ngaoundere-A1-F17 | *S. bovis* | [MH647131](https://www.ncbi.nlm.nih.gov/nucleotide/MH647131.1?report=genbank&log$=nucltop&blast_rank=14&RID=XF947NTT01N) | Cows | - | Cameroon | 1050 |
| Ngaoundere-A2-M21 | *S. bovis* | [MH647132](https://www.ncbi.nlm.nih.gov/nucleotide/MH647132.1?report=genbank&log$=nucltop&blast_rank=16&RID=XF9DE7JX01N) | Cows | - | Cameroon | 1050 |
| Ngaoundere-A2-M23 | *S. bovis* | [MH647133](https://www.ncbi.nlm.nih.gov/nucleotide/MH647133.1?report=genbank&log$=nucltop&blast_rank=9&RID=XF9DE7JX01N) | Cows | - | Cameroon | 1050 |
| Ngaoundere-A2-F24 | *S. bovis* | [MH647134](https://www.ncbi.nlm.nih.gov/nucleotide/MH647134.1?report=genbank&log$=nucltop&blast_rank=15&RID=XF9DE7JX01N) | Cows | - | Cameroon | 1050 |
| Ngaoundere-A3-F31 | *S. bovis* | MH647135 | Cows | - | Cameroon | 1050 |
| Ngaoundere-A3-F32 | *S. bovis* | [MH647136](https://www.ncbi.nlm.nih.gov/nucleotide/MH647136.1?report=genbank&log$=nucltop&blast_rank=12&RID=XF947NTT01N) | Cows | - | Cameroon | 1050 |
| Ngaoundere-A3-M37 | *S. bovis* | [MH6471](https://www.ncbi.nlm.nih.gov/nucleotide/MH647143.1?report=genbank&log$=nucltop&blast_rank=7&RID=XF9DE7JX01N)38 | Cows | - | Cameroon | 1050 |
| Ngaoundere-A13-M18 | *S. bovis* | [MH6471](https://www.ncbi.nlm.nih.gov/nucleotide/MH647143.1?report=genbank&log$=nucltop&blast_rank=7&RID=XF9DE7JX01N)42 | Cows | - | Cameroon | 1050 |
| Ngaoundere-A14-F22 | *S. bovis* | [MH647143](https://www.ncbi.nlm.nih.gov/nucleotide/MH647143.1?report=genbank&log$=nucltop&blast_rank=7&RID=XF9DE7JX01N) | Cows | - | Cameroon | 1050 |
| Ngaoundere-A14-F24 | *S. bovis* | MH647144 | Cows | - | Cameroon | 1050 |
| Ngaoundere-A14-M27 | *S. bovis* | MH647146 | Cows | - | Cameroon | 1050 |
| Ngaoundere-A16-M46 | *S. bovis* | [MH647148](https://www.ncbi.nlm.nih.gov/nucleotide/MH647148.1?report=genbank&log$=nucltop&blast_rank=30&RID=XF9DE7JX01N) | Cows | - | Cameroon | 1050 |
| Maroua-A19-F71 | *S. bovis* | MH647150 | Cows | - | Cameroon | 1050 |
| Maroua-A19-M76 | *S. bovis* | [MH647151](https://www.ncbi.nlm.nih.gov/nucleotide/MH647151.1?report=genbank&log$=nucltop&blast_rank=9&RID=XF947NTT01N) | Cows | - | Cameroon | 1050 |
| Maroua-A19-M77 | *S. bovis* | [MH647152](https://www.ncbi.nlm.nih.gov/nucleotide/MH647152.1?report=genbank&log$=nucltop&blast_rank=29&RID=XF9DE7JX01N) | Cows | - | Cameroon | 1050 |
| Maroua-A8-F81 | *S. bovis* | MH647153 | Cows | - | Cameroon | 1050 |
| Maroua-A8-F82 | *S. bovis* | MH647154 | Cows | - | Cameroon | 1050 |
| Maroua-A8-M86 | *S. bovis* | MH647155 | Cows | - | Cameroon | 1050 |
| Maroua-A8-M87 | *S. bovis* | MH647156 | Cows | - | Cameroon | 1050 |
| Maroua-A7-M77 | *S. bovis* | [MH647157](https://www.ncbi.nlm.nih.gov/nucleotide/MH647157.1?report=genbank&log$=nucltop&blast_rank=1&RID=XF947NTT01N) | Cows | - | Cameroon | 1050 |
| Bertoua-A4-M46 | *S. bovis* | [MH647160](https://www.ncbi.nlm.nih.gov/nucleotide/MH647160.1?report=genbank&log$=nucltop&blast_rank=23&RID=XF9DE7JX01N) | Cows | - | Cameroon | 1050 |
| Bertoua-A6-F61 | *S. bovis* | [MH647162](https://www.ncbi.nlm.nih.gov/nucleotide/MH647162.1?report=genbank&log$=nucltop&blast_rank=22&RID=XF9DE7JX01N) | Cows | - | Cameroon | 1050 |
| Bertoua-A6-F62 | *S. bovis* | [MH647163](https://www.ncbi.nlm.nih.gov/nucleotide/MH647163.1?report=genbank&log$=nucltop&blast_rank=25&RID=XF947NTT01N) | Cows | - | Cameroon | 1050 |
| Bertoua-A6-M65 | *S. bovis* | [MH647164](https://www.ncbi.nlm.nih.gov/nucleotide/MH647164.1?report=genbank&log$=nucltop&blast_rank=11&RID=XF9DE7JX01N) | Cows | - | Cameroon | 1050 |
| Garoua-A11-M126 | *S. bovis* | MH647165 | Cows | - | Cameroon | 1050 |
| Garoua-A10-F112 | *S. bovis* | [MH647169](https://www.ncbi.nlm.nih.gov/nucleotide/MH647169.1?report=genbank&log$=nucltop&blast_rank=20&RID=XF9DE7JX01N) | Cows | - | Cameroon | 1050 |
| Garoua-A10-M117 | *S. bovis* | [MH647170](https://www.ncbi.nlm.nih.gov/nucleotide/MH647170.1?report=genbank&log$=nucltop&blast_rank=19&RID=XF9DE7JX01N) | Cows | - | Cameroon | 1050 |
| Garoua-A12-F131 | *S. bovis* | [MH647171](https://www.ncbi.nlm.nih.gov/nucleotide/MH647171.1?report=genbank&log$=nucltop&blast_rank=7&RID=XF947NTT01N) | Cows | - | Cameroon | 1050 |
| Garoua-A20-F81 | *S. bovis* | MH647173 | Cows | - | Cameroon | 1050 |
| Garoua-A20-F82 | *S. bovis* | [MH647174](https://www.ncbi.nlm.nih.gov/nucleotide/MH647174.1?report=genbank&log$=nucltop&blast_rank=10&RID=XF9C4FU901N) | Cows | - | Cameroon | 1050 |
| Garoua-A21-F91 | *S. bovis* | [MH647175](https://www.ncbi.nlm.nih.gov/nucleotide/MH647175.1?report=genbank&log$=nucltop&blast_rank=17&RID=XF9C4FU901N) | Cows | - | Cameroon | 1050 |
| Garoua-A21-M96 | *S. bovis* | MH647176 | Cows | - | Cameroon | 1050 |
| Garoua-A22-M102 | *S. bovis* | [MH647178](https://www.ncbi.nlm.nih.gov/nucleotide/MH647178.1?report=genbank&log$=nucltop&blast_rank=1&RID=XF9XP3PC016) | Cows | - | Cameroon | 1050 |
| Garoua-A22-F108 | *S. bovis* | [MH647179](https://www.ncbi.nlm.nih.gov/nucleotide/MH647179.1?report=genbank&log$=nucltop&blast_rank=22&RID=XF947NTT01N) | Cows | - | Cameroon | 1050 |
| V20M1_Rr_Hap2 | *S. bovis* | [MW022135](https://www.ncbi.nlm.nih.gov/nucleotide/MW022135.1?report=genbank&log$=nucltop&blast_rank=36&RID=XF947NTT01N) | *Rattus rattus* | Vekky | Benin | 1045 |
| V20M2_Rr_Hap3 | *S. bovis* | [MW022136](https://www.ncbi.nlm.nih.gov/nucleotide/MW022136.1?report=genbank&log$=nucltop&blast_rank=32&RID=XF9DE7JX01N) | *Rattus rattus* | Vekky | Benin | 1045 |
| V20M3_Rr_Hap4 | *S. bovis* | MW022137 | *Rattus rattus* | Vekky | Benin | 1045 |

C)

| **Haplotype/**  **isolate** | **Organism** | **GenBank**  **Acc.No.** | **Host** | **Locality** | **Country** | **Length**  **(bp)** |
| --- | --- | --- | --- | --- | --- | --- |
| 2758 | *S. guineensis* | AJ519517 | - | San Antonio | São Tomé and Principe | 1224 |
| tdSchGuin1 | *S. guineensis* | OX103896 | - | - | São Tomé and Principe | 1806 |
| 1970 | *S. guineensis* | AJ519523 | - | Edea | Cameroon | 1224 |
| - | *S. guineensis* | MK341584 | - | - | - | 1806 |

D)

| **Haplotype/**  **isolate** | **Organism** | **GenBank**  **Acc.No.** | **Host** | **Locality** | **Country** | **Length**  **(bp)** |
| --- | --- | --- | --- | --- | --- | --- |
| 2797 | *S. mansoni* | AJ519524 | - | - | Senegal | 1224 |
| Sm_Coi1 | *S. mansoni* | MG562512 | *Homo sapiens* | - | Côte d'Ivoire | 1029 |
| Sm_Coi5 | *S. mansoni* | MG562513 | *Homo sapiens* | - | Côte d'Ivoire | 1029 |
| GCA_000237925 | *S. mansoni* | [HE601612](https://www.ncbi.nlm.nih.gov/nucleotide/HE601612.2?report=genbank&log$=nucltop&blast_rank=1&RID=2V2709K9013) | *-* | - | Puerto Rico | 1806 |
| - | *S. mansoni* | [NC_002545](https://www.ncbi.nlm.nih.gov/nucleotide/NC_002545.1?report=genbank&log$=nucltop&blast_rank=2&RID=2V2709K9013) | *-* | - | - | 1532 |
| - | *S. mansoni* | [AF101196](https://www.ncbi.nlm.nih.gov/nucleotide/AF101196.1?report=genbank&log$=nucltop&blast_rank=3&RID=2V2709K9013) | *-* | - | Puerto Rico | 1876 |

Supplementary TABLE S4: Distribution of the *cox*1 sequences analyzed for haplotype networks of: A) *S. haematobium (Sh);* B) *S. bovis (Sb); and* C) *S. mansoni (Sm),* and the corresponding haplotypes they provided, according to their country and host. H, haplotype.

A)

| Haplotype  (nº samples) | Haplotype/isolate code | GenBank  Acc.No. | Country | Host |
| --- | --- | --- | --- | --- |
| H1 (8) | Sh-*cox*1-H1  G1  Pointe Noire  - | PP905315  PP905315  PP905315  PP905315  PP905315  MK253577  KY967520  KT354660 | Guinea Bissau  Côte d'Ivoire  Mali  Mauritania  Senegal  Tanzania  Rep. Congo  Gabon | Human  Human  Human  Human  Human  Human  Human  *M. auratus* |
| H2 (2) | Sh-*cox*1-H2 | PP905316  PP905316 | Gambia  Mauritania | Human |
| H3 (1) | Sh-*cox*1-H3 | PP905317 | Mali | Human |
| H4 (1) | Sh-*cox*1-H4 | PP905318 | Mali | Human |
| H5 (1) | Sh-*cox*1-H5 | PP905319 | Senegal | Human |
| H6 (1) | Sh-*cox*1-H6 | PP905320 | Mali | Human |
| H7 (1) | Sh-*cox*1-H7 | PP905321 | Mali | Human |
| H8 (1) | Sh-*cox*1-H8 | PP905322 | Mali | Human |
| H9 (2) | Sh-*cox*1-H9 | PP905323  PP905323 | Mali  Guinea Bissau | Human Human |
| H10 (1) | Sh-*cox*1-H10 | PP905324 | Mali | Human |
| H11 (1) | Sh-*cox*1-H11 | PP905325 | Mali | Human |
| H12 (1) | Sh-*cox*1-H12 | PP905326 | Mali | Human |
| H13 (1) | Sh-*cox*1-H13 | PP905327 | Mali | Human |
| H14 (2) | Sh-*cox*1-H14  - | PP905328  KT354661 | Senegal  Benin | Human  *M. auratus* |
| H15 (3) | Sh-*cox*1-H15  - | PP905329  PP905329  NC_008074 | Mali  Senegal  Mali | Human  Human  *M. auratus* |
| H16 (1) | Sh-*cox*1-H16 | PP905330 | Senegal | Human |
| H17 (1) | Sh-*cox*1-H17 | PP905331 | Mali | Human |
| H18 (1) | Sh-*cox*1-H18 | PP905332 | Côte d'Ivoire | Human |
| H19 (1) | Sh-*cox*1-H19 | PP905333 | Mali | Human |
| H20 (3) | Sh-*cox*1-H20 | PP905334 | Mali | Human |
| H21 (1) | Sh-*cox*1-H21 | PP905335 | Senegal | Human |
| H22 (2) | Sh-*cox*1-H22  tdSchHaem1.1 | PP905336  OX104052 | Mali  Egypt | Human  - |
| H23 (1) | Sh-*cox*1-H23 | PP905337 | Mauritania | Human |
| H24 (1) | Sh-*cox*1-H24 | PP905338 | Mali | Human |
| H25 (1) | Sh-*cox*1-H25 | PP905339 | Mali | Human |
| H26 (1) | Sh-*cox*1-H26 | PP905340 | Senegal | Human |
| H27(1) | Sh-*cox*1-H27 | PP905341 | Senegal | Human |
| H28 (1) | Sh-*cox*1-H28 | PP905342 | Mali | Human |
| H29 (1) | Sh-*cox*1-H29 | PP905343 | Mali | Human |
| H30 (1) | Sh-*cox*1-H30 | PP905344 | Mali | Human |
| H31 (1) | Sh-*cox*1-H31 | PP905345 | Senegal | Human |
| H32 (1) | Sh-*cox*1-H32 | PP905346 | Mali | Human |
| H33(1) | Sh-*cox*1-H33 | PP905347 | Mali | Human |
| H34 (1) | Sh-*cox*1-H34 | PP905348 | Mali | Human |
| H35 (1) | G1 | MK253567 | Niger | Human |
| H36 (1) | LgHap1 | EU567127 | Malawi | *B. globosus* |
| H37 (1) | 3572 | AJ519520 | Senegal | - |

B)

| Haplotype  (nº samples) | Haplotype/isolate code | GenBank  Acc.No. | Country | Host |
| --- | --- | --- | --- | --- |
| H1 (1) | Sb-*cox*1-H1 | PP905349 | Mauritania | Human |
| H2 (1) | Sb-*cox*1-H2 | PP905350 | Mali | Human |
| H3 (1) | Sb-*cox*1-H3 | PP905351 | Senegal | Human |
| H4 (1) | Sb-*cox*1-H4 | PP905352 | Mali | Human |
| H5 (4) | Sb-*cox*1-H5  Sh_Corsica3 | PP905353  PP905353  PP905353  KT354658 | Mali  Senegal  Mauritania  Corsica, France | Human  Human  Human  Human |
| H6 (1) | Sh_Corsica1 | KT354656 | Corsica, France | Human |
| H7 (1) | Sh_Corsica2 | KT354657 | Corsica, France | Human |
| H8 (1) | B2 | AJ519521 | Senegal | - |
| H9 (1) | Ngaoundere-A1-M13 | MH647122 | Cameroon | Cows |
| H10 (1) | V20M1_Rr_Hap2 | MW022135 | Benin | *R. rattus* |
| H11 (1) | RT17_TMB4.5 | MT579448 | Senegal | *Bos indicus* |
| H12 (1) | RT16.1_DJc7.4 | MT579446 | Senegal | *B. truncatus* |
| H13 (1) | RT16.1_Th1.2 | MT579440 | Senegal | *B. truncatus* |

C)

| Haplotype  (nº samples) | Haplotype/isolate code | GenBank  Acc.No. | Country | Host |
| --- | --- | --- | --- | --- |
| H1 (3) | Sm-*cox*1-H1 | PP905356 | Senegal | Human |
| H2 (1) | Sm-*cox*1-H2 | PP905357 | Senegal | Human |
| H3 (1) | Sm-*cox*1-H3 | PP905358 | Senegal | Human |
| H4 (1) | Sm-*cox*1-H4 | PP905359 | Senegal | Human |
| H5 (1) | Sm-*cox*1-H5 | PP905360 | Senegal | Human |
| H6 (1) | Sm-*cox*1-H6 | PP905361 | Senegal | Human |
| H7 (1) | Sm-*cox*1-H7 | PP905362 | Senegal | Human |
| H8 (1) | Sm-*cox*1-H8 | PP905363 | Senegal | Human |
| H9 (1) | Sm-*cox*1-H9 | PP905364 | Guinea | Human |
| H10 (1) | Sm-*cox*1-H10 | PP905365 | Guinea | Human |
| H11 (2) | Sm-*cox*1-H11  Sm_Coi1 | PP905366  MG562512 | Guinea  Côte d'Ivoire | Human  Human |
| H12 (1) | Sm-*cox*1-H12 | PP905367 | Guinea | Human |
| H13 (1) | Sm-*cox*1-H13 | PP905368 | Guinea | Human |
| H14 (1) | Sm-*cox*1-H14 | PP905369 | Guinea | Human |
| H15 (1) | Sm-*cox*1-H15 | PP905370 | Guinea | Human |
| H16 (2) | Sm-*cox*1-H16  Sm_Coi5 | PP905371  MG562513 | Guinea  Côte d'Ivoire | Human |
| H17 (1) | Sm-*cox*1-H17 | PP905372 | Guinea | Human |
| H18 (1) | Sm-*cox*1-H18 | PP905373 | Côte d'Ivoire | Human |
| H19 (1) | Sm-*cox*1-H19 | PP905374 | Côte d'Ivoire | Human |
| H20 (1) | Sm-*cox*1-H20 | PP905375 | Côte d'Ivoire | Human |
| H21 (1) | E56_hsDJ | MN593380 | Senegal | Human |
| H22 (1) | E55_hsDJ | MN593376 | Senegal | Human |
| H23 (1) | E58_hsND | MN593384 | Senegal | Human |
| H24 (1) | E59_hsTE | MN593388 | Senegal | Human |
| H25 (1) | 2797 | AJ519524 | Senegal | - |
| H26 (1) | GCA_000237925 | HE601612 | Puerto Rico | - |
| H27 (1) | - | AF101196 | Puerto Rico | - |
